# Multifunctional MXene/Carbon Nanotube Janus Film for Electromagnetic Shielding and Infrared Shielding/Detection in Harsh Environments

**DOI:** 10.1007/s40820-024-01431-3

**Published:** 2024-06-14

**Authors:** Tufail Hassan, Aamir Iqbal, Byungkwon Yoo, Jun Young Jo, Nilufer Cakmakci, Shabbir Madad Naqvi, Hyerim Kim, Sungmin Jung, Noushad Hussain, Ujala Zafar, Soo Yeong Cho, Seunghwan Jeong, Jaewoo Kim, Jung Min Oh, Sangwoon Park, Youngjin Jeong, Chong Min Koo

**Affiliations:** 1 School of Advanced Materials Science and Engineering, Sungkyunkwan University, Seobu-ro 2066, Jangan-gu, Suwon-si, Gyeonggi-do 16419 Republic of Korea; 2 Department of Materials Science and Engineering, Soongsil University, Seoul, 06978 Republic of Korea; 3 Institute of Advanced Composite Materials, Korea Institute of Science and Technology, 92 Chudong-ro, Bongdong-eup, Wanju-gun, Jeollabuk-do 55324 Republic of Korea; 4 R&D Center INNOMXENE Co., Ltd., Daejeon, 34365 Republic of Korea; 5 School of Chemical Engineering, Sungkyunkwan University, Seobu-ro 2066, Jangan-gu, Suwon-si, Gyeonggi-do 16419 Republic of Korea

**Keywords:** MXene/carbon nanotube Janus film, Electromagnetic interference shielding, Infrared shielding, Thermal camouflage, Infrared detection

## Abstract

Multifunctional, flexible, and robust thin films capable of operating in demanding harsh temperature environments are crucial for various cutting-edge applications. This study presents a multifunctional Janus film integrating highly-crystalline Ti_3_C_2_T_*x*_ MXene and mechanically-robust carbon nanotube (CNT) film through strong hydrogen bonding. The hybrid film not only exhibits high electrical conductivity (4250 S cm^−1^), but also demonstrates robust mechanical strength and durability in both extremely low and high temperature environments, showing exceptional resistance to thermal shock. This hybrid Janus film of 15 μm thickness reveals remarkable multifunctionality, including efficient electromagnetic shielding effectiveness of 72 dB in X band frequency range, excellent infrared (IR) shielding capability with an average emissivity of 0.09 (a minimal value of 0.02), superior thermal camouflage performance over a wide temperature range (− 1 to 300 °C) achieving a notable reduction in the radiated temperature by 243 °C against a background temperature of 300 °C, and outstanding IR detection capability characterized by a 44% increase in resistance when exposed to 250 W IR radiation. This multifunctional MXene/CNT Janus film offers a feasible solution for electromagnetic shielding and IR shielding/detection under challenging conditions.

**Supplementary Information:**

The online version contains supplementary material available at 10.1007/s40820-024-01431-3.

## Highlights

- A multifunctional Janus film is fabricated by integrating highly-crystalline and oxidation-resistant Ti_3_C_2_T_*x*_ MXene with carbon nanotube (CNT) film through strong hydrogen bonding, which exhibits high electrical conductivity of 4250 S cm^−1^ and robust mechanical strength of 77 MPa.
- The MXene/CNT Janus film of 15 μm thickness demonstrates efficient electromagnetic interference shielding of 72 dB, low infrared (IR) emissivity of 0.09 and hence superior thermal camouflage performance, and outstanding IR detection capability, while maintaining its integrity equally at room temperature as well as under extreme conditions.
- This multifunctional MXene/CNT Janus film offers a practical solution for electromagnetic shielding and IR shielding/detection in challenging conditions.

**Supplementary Information:**

The online version contains supplementary material available at 10.1007/s40820-024-01431-3.

## Graphical abstract

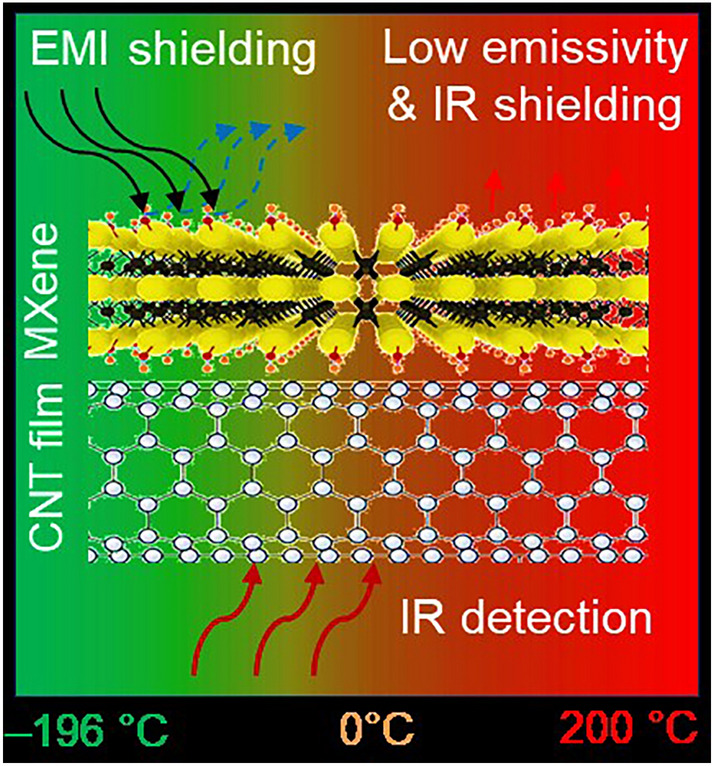

## Introduction

The need for multifunctional, flexible, robust, and ultrathin films has surged to meet the evolving requirements in electronics, thermal management, military, and aerospace technologies. However, bestowing these films with multifunctional characteristics such as electromagnetic interference (EMI) shielding and the capability to conceal and detect infrared (IR) radiation is particularly challenging under harsh environmental conditions involving extremely low and high temperatures [1–8].

Conventional metals such as Cu and Al exhibit superior EMI and IR-shielding capabilities owing to their highly conductive nature and low IR emissivity [9–14]. However, their high density, processing complexity, and susceptibility to corrosion in harsh environments limit their practical use [11, 15, 16]. Carbon-based materials such as carbon nanotubes (CNTs) and graphene have emerged as promising alternatives to EMI-shielding IR-detecting materials, owing to their excellent mechanical strength and IR-absorbing capabilities [14, 17–19]. However, their moderate electrical conductivity requires the use of thicker films for achieving satisfactory EMI shielding [20–22] and their high IR emissivity impairs their utility for IR shielding and thermal camouflage applications [22].

Recently, two-dimensional transition metal carbides/nitrides (also known as MXenes) have garnered attention as multifunctional nanomaterials owing to their excellent metallic conductivity, low IR emissivity, tunable composition and surface chemistry, and outstanding solution processibility for various applications including EMI shielding, energy storage, sensing, and thermal camouflage [23–34]. These materials have the general formula M_*n*+1_X_n_T_*x*_ (*n* = 1–4), where M, X, and T_*x*_ represent a transition metal, carbon or nitrogen, and surface terminal groups (–OH/ = O/–F), respectively. However, despite their promising attributes, MXenes face challenges associated with their inferior mechanical properties and poor environmental stability under harsh conditions, primarily owing to their defective atomic structure, which restricts their practical applications [35].

MXenes are typically synthesized by selectively etching the element A from their parent MAX crystals using strong acids. However, they tend to contain atomic defects or vacancies along their edges and surfaces, which initiate oxidative degradation in the presence of water and/or oxygen, leading to the degradation of their physical properties [36–38]. In particular, the atomic defects in Ti-based MXenes (Ti_3_C_2_T_*x*_) function as active sites for Ti oxidation, with Ti vacancies acting as positively charged regions and C^4−^ ions losing electrons to promote the formation of TiO_2_ [39]. Recently, highly crystalline Al-rich Ti_3_AlC_2_ MAX crystals have been synthesized by introducing excess Al precursor. The reported process substantially reduced the defect density, enhanced the oxidation resistance, and improved the electrical conductivity by up to 20,000 S cm^−1^ [40]. However, the insufficient mechanical flexibility, strength, and durability of the MXenes—both at room temperature and under harsh environmental conditions—constrain their practical applications.

MXene-based polymer composites and hybrids have been extensively studied to improve the mechanical strength and durability of MXenes in harsh environments [41–50]. Hydrophilic terminal groups on the MXene surface, such as –OH, =O, and –F, facilitate the formation of strong hydrogen bond interactions with polar polymeric chains, thereby improving the mechanical strength and durability [44, 51–57]. However, non-conducting feature of polymers suppresses the electrical conductivity, thereby retarding the EMI-shielding as well as IR-shielding capabilities of MXenes, and increases their IR emissivity [24, 58]. Combining MXenes with carbon-based materials, such as CNT, graphene, and reduced graphene oxide (rGO), can result in mechanical properties and EMI-shielding capability superior to those of MXene–polymer composites, owing to the conductive nature of carbon materials [59]. However, this combination strategy significantly impairs the IR-shielding ability of MXenes, owing to the strong IR-absorbing capability of carbon materials, leading to higher emissivity [60]. Consequently, research is focusing on optimizing a composite structure that fully leverages the high conductivity, low IR emissivity, and impressive EMI-shielding capability of MXenes in addition to the mechanical robustness of carbon materials. In this regard, Janus structures, which exhibit different microstructures and chemical components on opposite sides, have recently gained prominence because of their tunable mechanical properties and synergistic multifunctionality, without sacrificing intrinsic properties [47, 61, 62]. However, existing literature lacks comprehensive coverage of MXene's challenges concerning oxidation and its inferior mechanical characteristics, as well as the retention of these properties under extreme temperature conditions. Furthermore, there also remains a significant gap in the exploration of the infrared shielding and detection functionalities, particularly in challenging environments.

In this study, a bi-layer Janus-type hybrid film composed of highly-conductive and environmentally-stable Al–Ti_3_C_2_T_*x*_ MXene coating on a robust CNT film was developed. The robust CNT film with an average thickness of 10 µm was prepared using an aerogel spinning method based on a modified chemical vapor deposition followed by roller pressing (calendaring). A highly crystalline Ti_3_C_2_T_*x*_ MXene (Al–Ti_3_C_2_T_*x*_) with improved electrical conductivity and environmental stability was synthesized by selectively etching of A elements from Ti_3_AlC_2_ MAX phase, which was synthesized with excess Al precursor. Subsequently, Janus films of Ti_3_C_2_T_*x*_ MXene/CNT (denoted as MC) were fabricated by coating the CNT film with delaminated Ti_3_C_2_T_*x*_ MXene sheets using various MXene/CNT weight ratios (1:4 (MC14), 1:2 (MC12), and 1:1 (MC11)) by vacuum-assisted filtration. The resulting Janus films exhibited outstanding mechanical strength and durability, as well as impressive multifunctional properties including superior EMI shielding and IR shielding/detection capabilities, even at extremely low and high temperatures, rendering it highly desirable for integrated electronics, thermal management systems, and military and aerospace applications.

## Experimental Section

### Materials and Chemicals

Conventional Ti_3_AlC_2_ MAX phase (99.9% purity) was purchased from INNOMXENE Co., Ltd., Republic of Korea. Powder of titanium oxide (TiO_2_, 99.5%, 400 mesh) was purchased from Junsei Chemical Co., Ltd. Lithium fluoride (LiF, 98.5%), Ti (99.5%, 325 mesh), graphite (99.8%, 325 mesh), and Al (99.5%, 325 mesh) powders were sourced from Alfa Aesar. Hydrochloric acid (HCl, 37%), hydrofluoric acid (HF, ACS reagent grade, 48%), ferrocene (0.2 wt%, 98%), polysorbate (1.0 wt%), and thiophene (0.8 wt%, 99%) were purchased from Sigma Aldrich. Acetone (98.0 wt%) and N-methyl-2-pyrrolidone (NMP) were procured from Samchun Chemical, Republic of Korea. The CNTs powder (97%, diameter 20 nm, and length 10 μm) were purchased from Applied Carbon Nano Technology, Republic of Korea. All chemicals were used as received.

### Synthesis of Al–Ti_3_AlC_2_ MAX and Al–Ti_3_C_2_T_*x*_ MXene

To synthesize highly crystalline Al-rich Al–Ti_3_AlC_2_ MAX, we synthesized the high-quality TiC powder through a high-energy ball milling-assisted carbothermal reduction method, employing titanium oxide (TiO_2_) and carbon powder with a molar ratio of TiO_2_:C = 1:2.4 [36]. The TiO_2_ and graphite powder mixture underwent high-energy ball milling in a planetary mill (OMBMNN-1507231, MTDI Inc., Korea). Subsequently, the powder mixture underwent heat-treatment at 1500 °C for 3 h under a vacuum of 6.7 × 10^**−**3^ Pa to synthesize a high-quality TiC powder. The synthesized TiC was mixed with Ti and accessed Al precursors in a molar ratio of 2:1.2:2.2 and subsequently subjected to continuous ball milling for 12 h at 100 rpm. The resulting mixture was molded into a Ø50 mm disk using a hydraulic press with 4000 psi, followed by thermal annealing at 1450 °C for 3 h in argon atmosphere at atmospheric pressure. The resulting Al–Ti_3_AlC_2_ MAX disk was pulverized using a jaw crusher, followed by washing with 9 M HCl for 12 h and rinsing with deionized water multiple times to remove impurities. Subsequently, the purified MAX powder was then dried at 60 °C for 12 h in a vacuum oven. The dried powder was sieved through a 270-mesh (53 µm) sieve and used for the synthesis of Al–Ti_3_C_2_T_*x*_ MXene.

The Al–Ti_3_C_2_T_*x*_ MXene was synthesized using a modified minimally intensive layer delamination (MILD) method, which involved selectively etching Al from the parent Al–Ti_3_AlC_2_ MAX phase [63]. Briefly, LiF (1.6 g) was dissolved in a mixture of HCl (20 mL, 9 M) and an aqueous HF solution (3 mL) in a polypropylene container and stirred. To prevent the sudden temperature increase caused by exothermic reaction, the Al–Ti_3_AlC_2_ MAX powder (1 g) was gradually added to the LiF/HCl/HF solution while stirring continuously with a magnetic stirrer. Under these conditions, the etching reaction was allowed to proceed for 24 h at 35 °C. The product obtained after etching was washed several times with deionized water by centrifugation at 3500 rpm for 5 min until the pH of the supernatant reached 5–6. Subsequently, the obtained delaminated Al–Ti_3_C_2_T_*x*_ flakes in an aqueous dispersion were used for sample preparation and characterization. For comparison, the conventional Ti_3_C_2_T_*x*_ MXene was synthesized using the Ti_3_AlC_2_ MAX phase (InnoMXene) by following the same synthesis protocol.

### Synthesis of CNT Film

CNT film was synthesized through a modified chemical vapor deposition method [64]. In this synthesis, a precursor solution was prepared by mixing ferrocene (Fe(C_5_H_5_)_2_, 0.2 wt%, 98% purity), thiophene (C_4_H_4_S, 0.8 wt%, 99% purity), (C_3_H_6_O, 98.0%, 99.7% purity), and polysorbate (1 wt%) [65, 66]. The resulting mixture was injected into a vertically oriented reactor heated to 1250 °C at a rate of 10 mL h^**−**1^. Hydrogen carrier gas (H_2_) was introduced at a flow rate of 1000 sccm. During the synthesis, ferrocene decomposed into catalyst iron nanoparticles at 400 °C [67]. As the reactor temperature ranged between 800 and 900 °C, thiophene degraded into sulfur, acting as a catalyst activator and combining with iron to create iron sulfide [67]. Subsequently, carbon from acetone diffused into iron sulfide, initiating the nucleation of carbon nanotube. The carbon nanotubes grew and integrated into an aerogel CNT sock, continuously winding onto a cylindrical winder, ultimately producing a CNT film at the bottom of the reactor. The modified chemical vapor deposition process allows for adjustable thickness of the CNT film, ranging sub-micron to as thick as required. In this study, the synthesized CNT film with an average thickness of 10 µm and a large area of 100 × 300 cm^2^ was used throughout the experiments.

### Fabrication of MXene/CNT (MC) *Janus* Film

The CNT sheet was subjected to UV–ozone treatment (UV–Ozone cleaner AC-3) for 5 min to introduce hydrophilic oxygen-containing functional groups on its surface. The ozone-treated CNT sheet was then cut into a circular shape with the same diameter as that of the filtration flask (38 mm) and placed on a polyvinylidene fluoride filter membrane (pore size, 0.45 µm). Subsequently, an Al–Ti_3_C_2_T_*x*_ MXene dispersion (4 mg mL^−1^) was poured on top of the CNT film at different loading concentrations to obtain Janus films with different MXene/CNT (MC) mass ratios (1:4, 1:2, and 1:1; denoted as MC14, MC12, and MC11, respectively). The resulting MC Janus films were vacuum dried at 100 °C for 8 h. The MXene and CNT sides of the Janus films are denoted as MC-M and MC-C, respectively. For comparison, an Al–Ti_3_C_2_T_x_ MXene/CNT (1:1) blend film (MC11-Blend) was prepared by simply mixing the Al–Ti_3_C_2_T_*x*_ MXene and multiwalled CNT (with a diameter of 20 nm) powder, followed by vacuum filtration.

### Characterization

Surface and cross-sectional morphologies of the MXene flakes, CNT sheets, and MC Janus films were examined by field-emission SEM (JSM-7600F, Japan) coupled with energy dispersive spectroscopy (EDS). The crystal structure was investigated by XRD (D8, Bruker, USA) using Cu Kα radiation. The XRD scans covered a 2*θ* range of 4°–80° at a rate of 2° min^**−**1^, with a window slit dimension of 10 × 10 mm^2^. For the atomic-scale structural analysis, HRTEM (JEM-2100F, Japan) was performed at an acceleration voltage of 200 kV to obtain high-resolution images of individual MXene flakes. To investigate the elemental compositions of the specimens, XPS was performed using Al Kα radiation (1486.6 eV) with an ESCALAB250 (USA) instrument. FTIR spectroscopy (JASCO-4700, Japan) was conducted to identify and analyze the chemical compositions of the materials based on their interactions with IR light. Electrical conductivity was measured using a four-pin probe (MCP-TP06P PSP) connected to a Loresta-GP meter (Model MCP-T610, Mitsubishi Chemical, Japan). EMI-shielding measurements were performed using a two-port network analyzer (ENA5071C, Agilent Technologies, USA) with a rectangular WR-90 waveguide in TE10 mode in the X-band (frequency range of 8.2–12.4 GHz), and Ku band (frequency range 12.4–18 GHz).

The mechanical properties were explored at ambient, low (− 65 °C), and high temperatures (200 °C) using a universal testing machine (Instron 5567A) having environmental chamber with a crosshead speed of 5 mm min^−1^ and a 2 KN load cell. Each specimen was rectangular and had a length, width, and variable thickness of 30 mm, 10 mm, and 10–17 µm, respectively. Each sample underwent tensile testing five times.

IR images and temperature data were acquired using an IR camera (FLIR A310, FLIR, Sweden) with an emissivity of 0.94. An FTIR spectrometer (JASCO-4700, Japan) was employed to determine the reflectivity (*R*) of the MXene coatings in the 2.5–15 μm range at room temperature. The reflectance of the thin film was directly measured, and the emissivity *ε* was calculated using Kirchhoff’s law as ε = 1 − *R*.

The performance of the IR detector was evaluated using a digital multimeter (Keysight 34450A). Essentially, an IR lamp (HH2500) was used to illuminate the MC11-C sample measuring 2 cm × 2.5 cm, and the resulting changes in resistance and temperature were measured using the digital multimeter. The water contact angles of the MXene thin films were determined using a camera (UI-1220LE-M-GL, IDS, Germany).

## Results and Discussion

### Preparation and Characterization of Ti_3_C_2_T_x_/CNT *Janus* Films

**Fig. 1:**
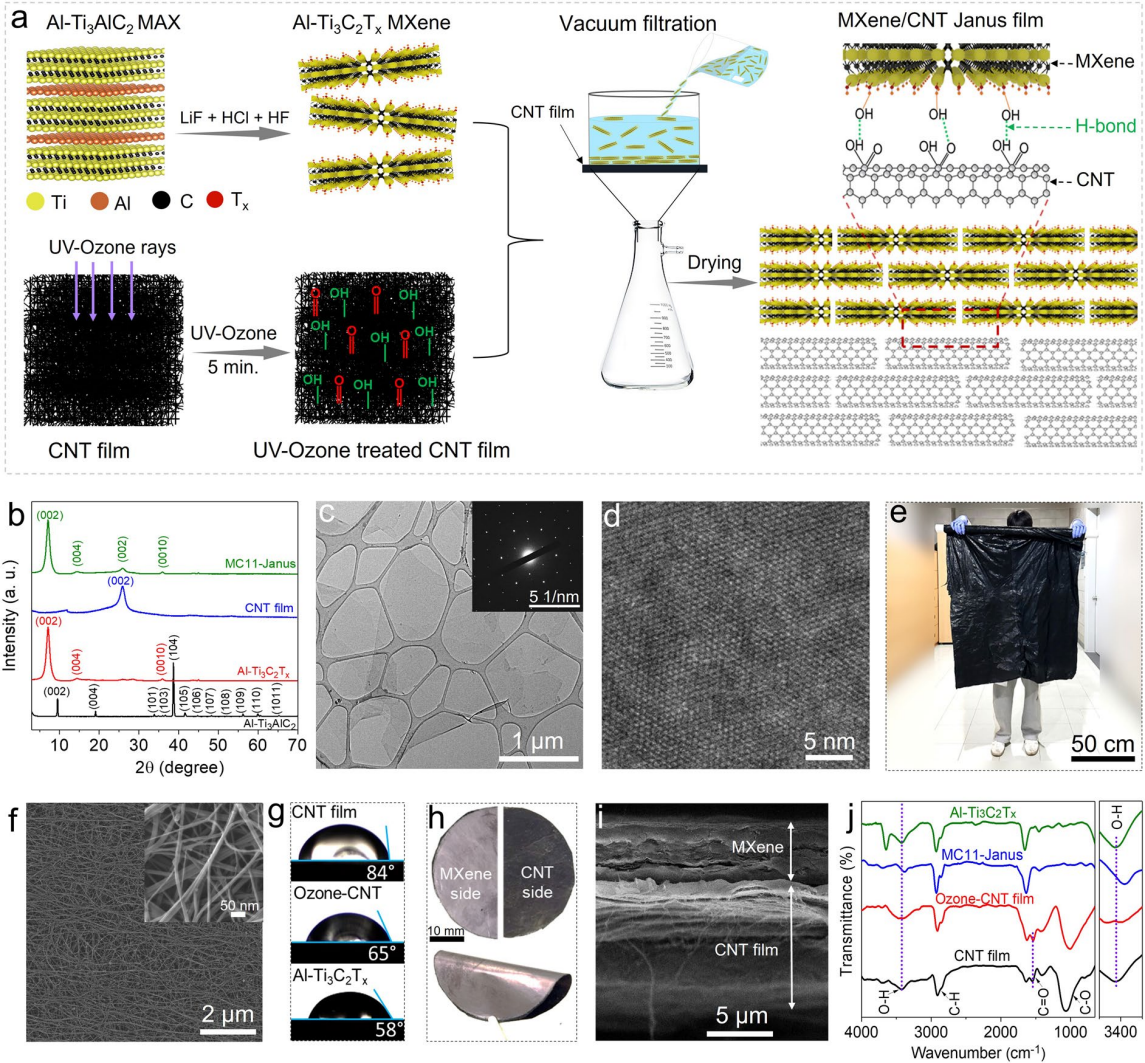
Synthesis and characterization of MXene/carbon nanotube (CNT) Janus film. **a** Schematic illustrating the Al–Ti_3_C_2_T_*x*_ MXene synthesis and MXene/CNT Janus film fabrication. **b** XRD patterns of the modified Al–Ti_3_AlC_2_ MAX phase, highly crystalline Al–Ti_3_C_2_T_*x*_ MXene, CNT film, and MC11 Janus film **c** TEM image of a single Al–Ti_3_C_2_T_*x*_ flake (inset: SAED pattern). **d** HRTEM image of Al–Ti_3_C_2_T_*x*_, indicating its highly crystalline atomic structure. **e** Prepared CNT film with a large size held by a volunteer. **f** Low-magnification SEM image of the CNT film, obtained to examine its surface morphology (inset: high-magnification image). **g** Water contact angles of the CNT, ozone-treated CNT, and Al–Ti_3_C_2_T_*x*_ MXene films, highlighting the ozone-treatment-induced increase in the hydrophilicity of the CNT surface. **h** Photographs of the CNT and MXene sides of the remarkably flexible Janus-type hybrid film. **i** Cross-sectional SEM image of the MC11 film, confirming its bilayer Janus-type nature. **j** FTIR spectra of the CNT, ozone-treated CNT, MC11 Janus, and Al–Ti_3_C_2_T_*x*_ MXene films

The MC Janus film was prepared by sequentially depositing Al–Ti_3_C_2_T_*x*_ MXene onto a CNT film by vacuum-assisted filtration, which formed strong hydrogen bonds between the oxygen-containing functional groups at the interfaces (Fig. 1a). The Al–Ti_3_C_2_T_*x*_ MXene synthesis was validated by the shift of (002) X-ray diffractometry (XRD) peak toward a lower angle from 9.7**°** to 6.95**°**, indicating an increase in the interlayer spacing from 0.91 to 1.26 nm by the selective chemical etching of Al from the parent Ti_3_AlC_2_ MAX phase (red profile in Fig. 1b) [63]. The obtained aqueous dispersion of synthesized Al–Ti_3_C_2_T_x_ MXene was greenish in color (Fig. S1a), where the Scanning electron microscopy (SEM; Fig. S1b) and transmission electron microscopy (TEM) with selected area electron diffraction (SAED) analyses (Fig. 1c) of MXene flakes revealed their transparency and monolayer nature, which featured a smooth surface, sharp edges, and a maintained hexagonal lattice structure. High-resolution TEM (HRTEM) image of a single Al–Ti_3_C_2_T_*x*_ MXene flake (Fig. 1d) verified its highly crystalline atomic structure. The configured structure minimized Ti vacancies and oxygen impurities in the carbon sublattice, resulting in improved flake quality, which helped enhance the ensuing electrical conductivity and oxidative stability [36, 40]. Free-standing film of Al–Ti_3_C_2_T_*x*_ MXene was fabricated by vacuum filtration and its surface and cross-sectional morphologies, along with elemental compositions were examined by SEM (Fig. S1c–e). X-ray photoelectron spectroscopy (XPS) profiles demonstrate the successful elimination of Al 2*p* peak at 74.5 eV (Fig. S2a). The deconvoluted Ti 2*p* (Fig. S2b) peaks reveal that four pairs of doublets at 454.6 eV (460.9 eV), 455.6 eV (462.0 eV), 456.5 eV (463.3 eV), and 458.0 eV (465.3 eV) are assigned to the Ti–C, Ti^2+^, Ti^3+^, and Ti^4+^ (TiO_2_) respectively [38, 68]. The C 1*s* profile displays two major peaks at 282.2 and 284.4 eV, assigned to Ti–C and C–C respectively (Fig. S2c), and the elemental composition is shown in Fig. S2d [38, 68]. The pronounced Ti–C peak in both Ti 2*p*, and C 1*s* corroborated successful synthesis of Al–Ti_3_C_2_T_*x*_ MXene. For comparison, a conventional Ti_3_C_2_T_x_ MXene was synthesized from the Ti_3_AlC_2_ MAX phase using a stoichiometric ratio of Al precursor, following the aforementioned procedure (Fig. S3a–c). The UV–vis spectra with time indicates that Al–Ti_3_C_2_T_*x*_ MXene shows excellent oxidation stability as compared to the conv. Ti_3_C_2_T_*x*_ due to its highly crystalline atomic structure (Fig. S4a, b).

A conductive, robust, and large-area CNT film was fabricated using an aerogel spinning technique based on modified chemical vapor deposition, followed by roll pressing (Figs. 1e and S5) [64]. The presence of (002) peak at 2*θ* value of 26.1° is attributed to the CNT film, which is similar to the less-defective stacked neighboring graphene sheets (JCPDS 26-1079) (blue profile in Fig. 1b) [69, 70]. The CNT film had an average thickness of 10 µm over a large area of 100 × 300 cm^2^ (Fig. S6a). Low- and high-magnification SEM analyses of the CNT film indicated that individual CNT with an average diameter of approximately 21 nm (Fig. S6b) were aligned in the in-plane direction (Fig. 1f). The deconvoluted XPS of C 1*s* profile of the CNT film exhibited the presence of strong C–C peak at 284.8 eV, and the absence of peaks associated with oxygen impurities (e.g., C=O, and C–OH), indicating its high purity and excellent quality (Fig. S6c). The pristine CNT film had a hydrophobic surface with a water contact angle of 84° (Fig. 1g, Movie S1), presumably owing to its pure graphitic *sp*^2^ carbon bonds [71]. However, this led to poor compatibility with the hydrophilic surface terminations of Al–Ti_3_C_2_T_*x*_. To overcome this, the CNT film was subjected to UV–ozone treatment for 5 min to introduce enough oxygen-containing functional polar groups (–COOH, –OH, and C=O). Consequently, UV–ozone treated film showed improved hydrophilicity (contact angle, 65°), comparable to that of the Al–Ti_3_C_2_T_*x*_ MXene film (contact angle, 58°) (Fig. 1g, Movie S2) [72, 73]. Moreover, the EDS analysis shows increase in oxygen content from 3.5% for untreated CNT, to 9.1% for ozone treated CNT film (Figure S6d-e).

The flexible Janus films, comprising the Al–Ti_3_C_2_T_*x*_ MXene and ozone-treated CNT film, were fabricated by vacuum-assisted filtration with varying MXene content, followed by drying in a vacuum oven at 100 °C for 12 h, and analyzed for their XRD pattern (green profile in Fig. 1b, h). Cross-sectional SEM image (Fig. 1i) revealed a 15-μm-thick MC11 Janus film with robust integration between the two distinct layers, suggesting strong interlayer interactions. The respective EDS mapping (Fig. S7) confirmed the distribution of Ti and C, which primarily originated from Al–Ti_3_C_2_T_*x*_ MXene and CNT, respectively, thereby validating the formation of a Janus-type layered structure.

The interfacial interactions between Al–Ti_3_C_2_T_*x*_ MXene and CNT film were substantiated by Fourier-transform infrared (FTIR) spectroscopy. The FTIR spectrum of as-prepared CNT film showed peaks at 3430 cm^−1^ (O–H) and at 1580 and 1405 cm^−1^ (C=O) (black spectrum in Fig. 1j). After ozone treatment, a substantial increase in the intensity and broadness of the –OH and C=O peaks was observed, manifesting increased content of oxygen-containing functional groups; consistent with the decreased water contact angle. The Al–Ti_3_C_2_T_*x*_ MXene displayed characteristic peaks at 3430 and 1630 cm^−1^, corresponding to –OH vibrations of surface functional groups and adsorbed water, while the peak at 2920 cm^−1^ corresponds to C–H stretching [74–78]. Notably, the MC11 Janus film showed slight shift in the hydroxyl peak from 3432 to 3403 cm^−1^ compared with that of the as-prepared CNT and MXene films, indicating the formation of hydrogen bond interactions between the surface terminations of MXene (–OH and =O) and the oxygen-containing functional groups of the ozone-treated CNTs (magnified –OH peak in Fig. 1j) [61, 79]. The hydrogen bond interactions resulted in robust structural integration of the MXene–CNT Janus film. Hence, large-area MXene/CNT Janus films could also be fabricated using different processing techniques, such as spray coating, for potential applications. For comparison, a homogeneous composite film of Al–Ti_3_C_2_T_*x*_ MXene and CNT (denoted as MC11-Blend) was prepared by solution blending (Fig. S8a). The cross-sectional SEM and EDS results certified the distribution of MXene and CNTs throughout the MC11-Blend film (Fig. S8b–d).

### Mechanical and Electrical Properties Measurement

**Fig. 2:**
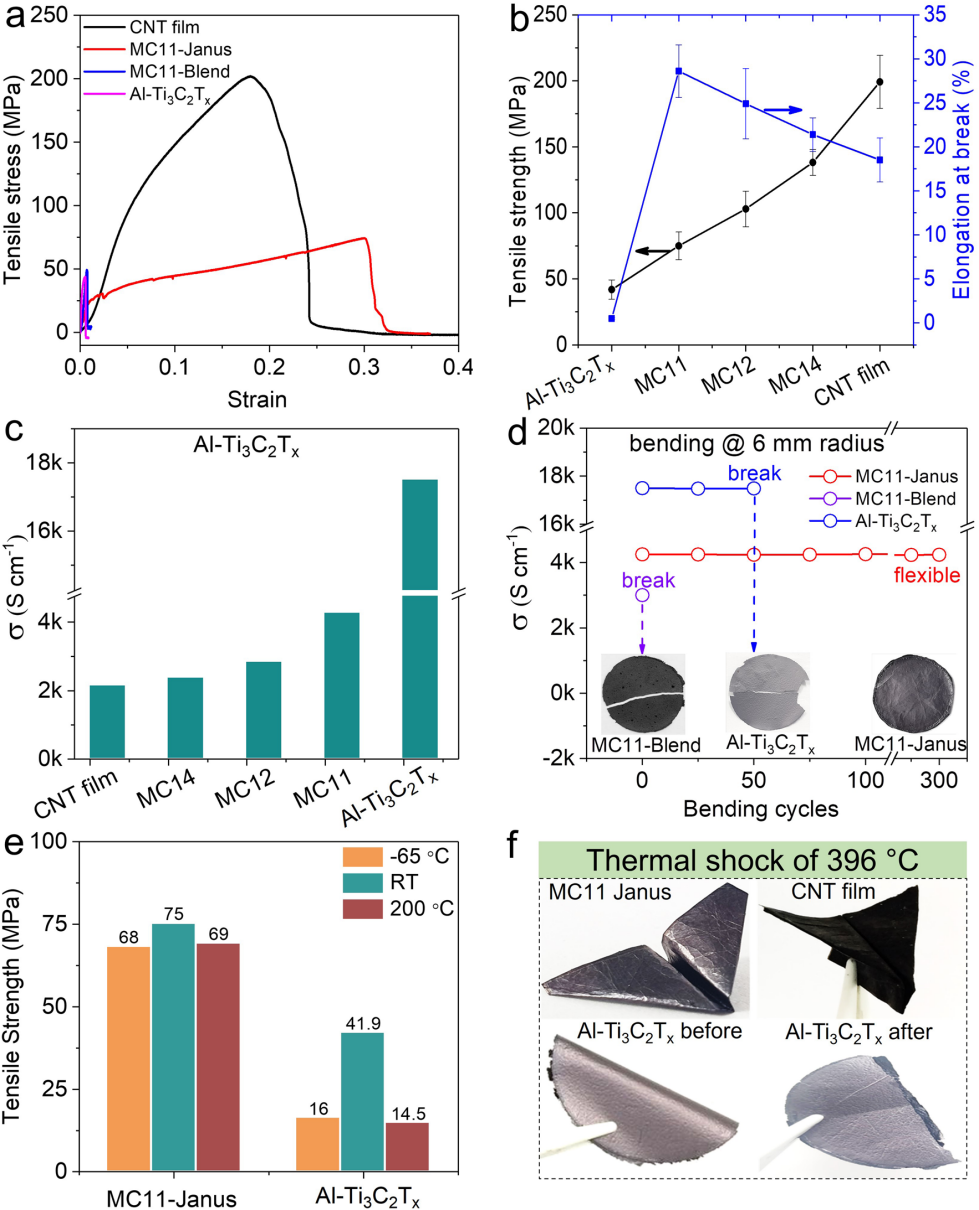
Mechanical strength and electrical conductivity. **a** Tensile stress–strain curves of the CNT, Al–Ti_3_C_2_T_*x*_ MXene, MC11-Janus, and MC11-Blend films. **b** Dependence of the tensile strength, and elongation at break values of the MC-Janus films on their composition at room temperature. **c** Electrical conductivities of the MC-Janus films with different compositions. **d** Electrical conductivities of the Al–Ti_3_C_2_T_*x*_ MXene, MC11-Janus, and MC11-Blend films after undergoing bending cycles with a 6 mm curvature (inset: flexibility of repeatedly bent films). **e** Abilities of the MC11-Janus and Al–Ti_3_C_2_T_*x*_ MXene films to retain mechanical strength at low and high temperatures (− 65 and 200 °C, respectively). **f** Flexibility of the CNT, MC11-Janus, and Al–Ti_3_C_2_T_*x*_ MXene films after undergoing a thermal shock of Δ396 °C ((exposure to liquid nitrogen (− 196 °C, 30 s) and an oven (200 °C, 30 s)) for 30 cycles

Mechanical flexibility, strength, and durability are crucial parameters for next-generation electronics, military, and aerospace applications, particularly under challenging environmental conditions, encompassing extremely low and high temperatures. Therefore, tensile stress–strain curves of the MC11 Janus, MC11-Blend, CNT, and Al–Ti_3_C_2_T_*x*_ MXene films were acquired at room temperature (Figs. 2a and S9a). The Al–Ti_3_C_2_T_*x*_ MXene film exhibited tensile strength and elongation-at-break values of 41 MPa and 0.5%, respectively. The MC11 Janus film demonstrated an improved elongation at break and tensile strength (77 MPa and 30%, respectively), primarily owing to the high tensile strength and elongation-at-break values of the CNT film (199.2 MPa and 18.5%). Notably, the MC11 Janus film exhibited considerably higher elongation at break and tensile strength values than those of the MC11-Blend film (48 MPa and 7%) of comparable thickness. It was also observed that the tensile strength improved with increasing CNT content (Fig. 2b). The Young's modulus of the Janus film also exhibited an increasing trend with the increase in MXene content due to its brittle nature (Fig. S9b). These results indicated that the enhanced strength and flexibility were due to the strong hydrogen bond interactions between the MXene and CNT layers, as well as the robustness of the CNT film.

Interestingly, electrical conductivity of Al–Ti_3_C_2_T_*x*_ MXene film (17,500 S cm^−1^) was double the value of conventional Ti_3_C_2_T_*x*_ MXene (8500 S cm^−1^), owing to its high crystallinity. The electrical conductivity of the Janus films decreased with increasing CNT content owing to relatively low conductivity of the CNT film (2130 S cm^−1^) (Fig. 2c and S10a). Therefore, MC11 Janus exhibited an electrical conductivity of 4250 S cm^−1^.

To assess mechanical flexibility, the MC11-Blend, MC11-Janus, and Al–Ti_3_C_2_T_*x*_ MXene films were subjected to bending cycles with a 6 mm curvature at room temperature (Fig. 2d), and evaluated for their retained electrical conductivities. The MC11-Blend film was highly brittle and hence, ruptured during the first bending cycle. The Al–Ti_3_C_2_T_*x*_ MXene film exhibited high electrical conductivity retention for 50 bending cycles but fractured thereafter. Notably, the MC11-Janus film showed efficient electrical conductivity retention for 300 bending cycles without any breakage, owing to its excellent flexibility. Furthermore, in tensile tests conducted at the extreme temperatures of − 65 and 200 °C, the MC11 Janus film showed marginal decreases in tensile strength (13% at − 65 °C and 12% at 200 °C), whereas the Al–Ti_3_C_2_T_*x*_ MXene film exhibited substantially reduced values (61% at − 65 °C and 65% at 200 °C; Fig. 2e). Essentially, owing to the excellent mechanical properties of the CNT and the formed hydrogen bonds, the Janus film retained its mechanical flexibility and electrical conductivity even under extreme environmental conditions [79]. Additionally, to assess the flexibility under high-temperature conditions, the CNT, MC11-Janus, and Al–Ti_3_C_2_T_*x*_ MXene films were subjected to a thermal shock of Δ396 °C (annealing in oven (200 °C, 30 s) followed by dipping into liquid nitrigen (− 196 °C, 30 s) Fig. 2f). Interestingly, the CM11-Janus film exhibited outstanding flexibility and durability even against severe thermal shocks. Also, the MC11-Janus film retained its flexibility even after exposure to liquid nitrogen and folding, in contrast to the Al–Ti_3_C_2_T_*x*_ MXene that became very brittle and ruptured (Movies S3–S5).

### Electromagnetic Shielding Effectiveness (SE) Measurement

**Fig. 3:**
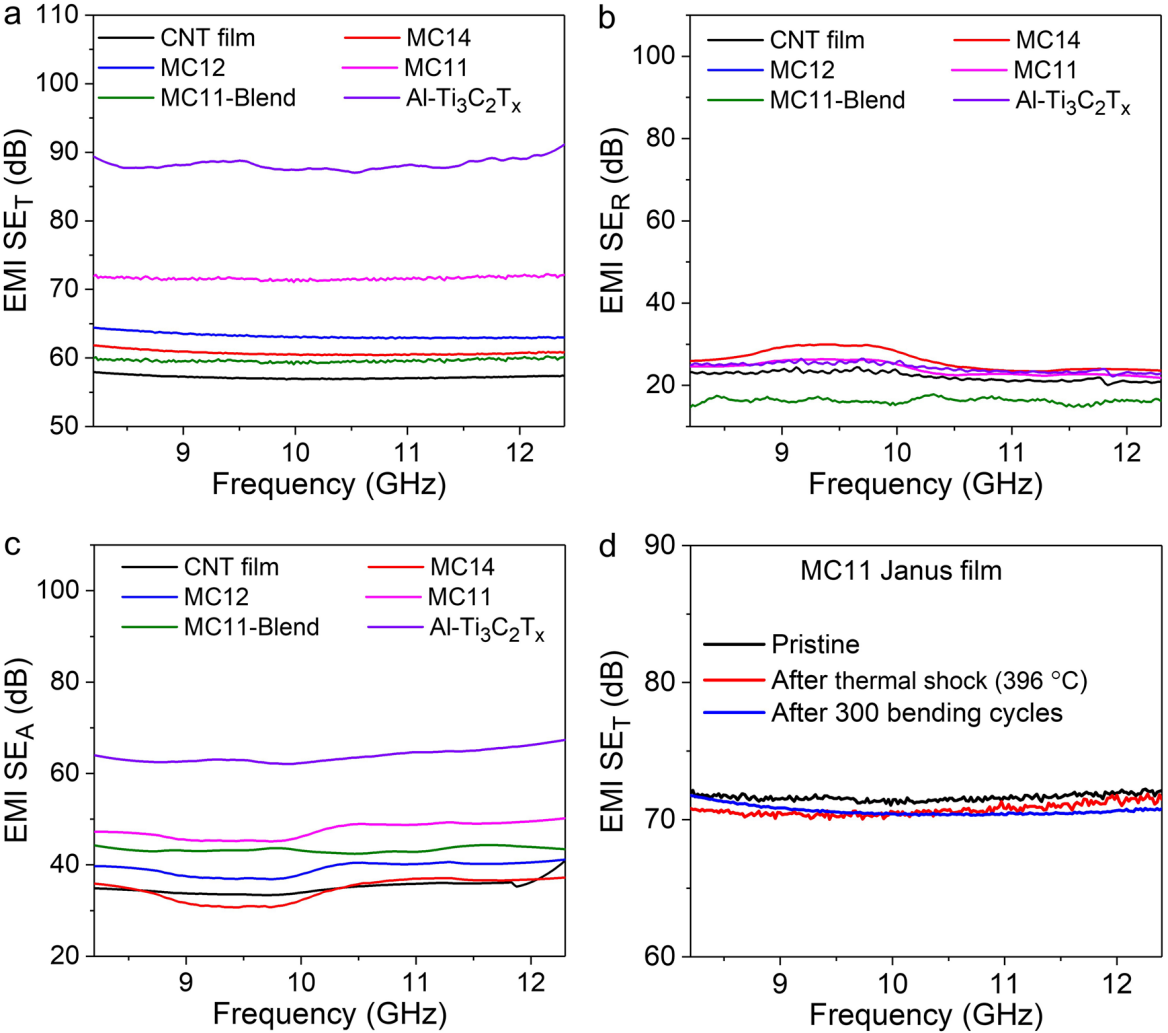
EMI-shielding capabilities of the MXene/CNT Janus film. **a** SE_T_ **b** SE_R_, and **c** SE_A_ value of the CNT film (10 µm), MC14 (11 µm), MC12 (12.5 µm), MC11 (15 µm), MC11-Blend (15 µm), and Al–Ti_3_C_2_T_*x*_ MXene (10 µm) film in X band. **d** Ability of the CM11-Janus film to retain its EMI-shielding attributes after thermal shock treatment of Δ396 °C for 30 cycles and 300 bending cycles with 6 mm curvature

The fabricated films of CNT, MC14, MC12, MC11-Janus, MC11-Blend, and Al–Ti_3_C_2_T_*x*_ were investigated for their total EMI-shielding effectiveness (SE_T_), shielding effectiveness by reflection (SE_R_), and shielding effectiveness by absorption (SE_A_) in the X-band frequency range of 8.2–12.4 GHz (Fig. 3a–c), and Ku band frequency range of 12.4–18 GHz (Fig. S12). A 10-µm-thick Al–Ti_3_C_2_T_*x*_ MXene film with the highest electrical conductivity exhibited higher SE_T_ value (88 dB) than that of the CNT and conventional Ti_3_C_2_T_*x*_ MXene films (57 and 73 dB, respectively). In the fabricated MC Janus films, the resulting EMI SET increased with increasing MXene content (and increased electrical conductivity), where the MC11 Janus film exhibited a value of 72 dB Fig. 3a. The conventional-MXene-based MC Janus films also showed a similar trend (Fig. S10b), however, the absolute values were lower than their counterparts. The SE_R_ values of all films saturated at approximately 25 dB (Fig. 3b), whereas the SE_A_ values of all the samples followed a trend similar to that of the SE_T_ values (Fig. 3c). Interestingly, the MC11 Janus film also exhibited outstanding specific shielding effectiveness (SSE/*t*) of 34,006 dB cm^2^ g^−1^ which is higher than most of the reported literature (Fig. S11 and Table S1). The large SSE/*t* value indicates that the fabricated MC11 Janus film have strong capability for potential use as lightweight and ultrathin EMI shielding material [34]. Furthermore, the SE_T,_ SE_R_, and SE_A_ values of all the samples have been analyzed in the Ku band, ranging from 12.4 to 18 GHz (Fig. S12a–c). The increasing trend in SE_T,_ and SE_A_ is observed for all samples in the Ku band compared to the X band, attributable to the increase in frequency. Conversely, the SE_R_ of all the samples exhibits a slight decrease in the Ku band. This behavior can be explained by Simon’s formulae for conductive materials, where SE_A,_ SE_R_ have a direct and inverse relation, respectively, to the frequency, as represented in the following equation:

$$SE_{T} = 50 + 10 {\text{log}} \left( {\frac{\sigma }{f}} \right) + 1.7{\text{ d}}\sqrt {\sigma f}$$

Here, σ, f, and d denote the electrical conductivity (S cm^-1^), frequency (MHz), and thickness (m), respectively. The first and second parts of the equation represent the shielding effectiveness reflection (SE_R_) and the shielding effectiveness absorption (SE_A_), respectively [80].

Additionally, the MC11-Janus film demonstrated significant retention of EMI-shielding capability after undergoing 300 bending cycles and 30 cycles of thermal shock with a temperature difference of Δ396 °C (Fig. 3d). Similarly, Al–Ti_3_C_2_T_*x*_ MXene exhibited good EMI-shielding retention, experiencing only a marginal decrease of 2 dB in EMI SE_T_, from 88 to 86 dB, after the large thermal shock of Δ396 °C. This minimal reduction was attributed to a slight decrease in electrical conductivity from 17,500 to 16,700 S cm^−1^. In contrast, conv.Ti_3_C_2_T_*x*_ MXene revealed relatively higher reduction in EMI shielding, dropping from 73 to 65 dB after the same thermal shock, manily due to a decrease in its electrical conductivity from 8500 to 5200 S cm^−1^ (Fig. S13). Therefore, these outstanding EMI shielding performance and its retention ability against large thermal shock were attributed to the excellent oxidative stability of the Al–Ti_3_C_2_T_*x*_ MXene, the outstanding mechanical flexibility of the CNT film, and the robust interfacial integration between the Al–Ti_3_C_2_T_*x*_ MXene and CNT films, even in harsh environments.

### Infrared Shielding and Thermal Camouflage

All the materials emit thermal radiation in the IR spectrum when their temperature exceeds absolute zero (0 K) [81]. According to the Stefan–Boltzmann law, expressed as *P* = *εσT*^*4*^ (where P, ε, σ, and T represent the thermal radiation emitted by an object, IR emissivity, Stefan–Boltzmann constant, and the surface-level thermodynamic temperature of the object, respectively), the thermal radiation emitted by an object is directly proportional to the fourth power of the surface temperature and the IR emissivity of the material [82].

**Fig. 4:**
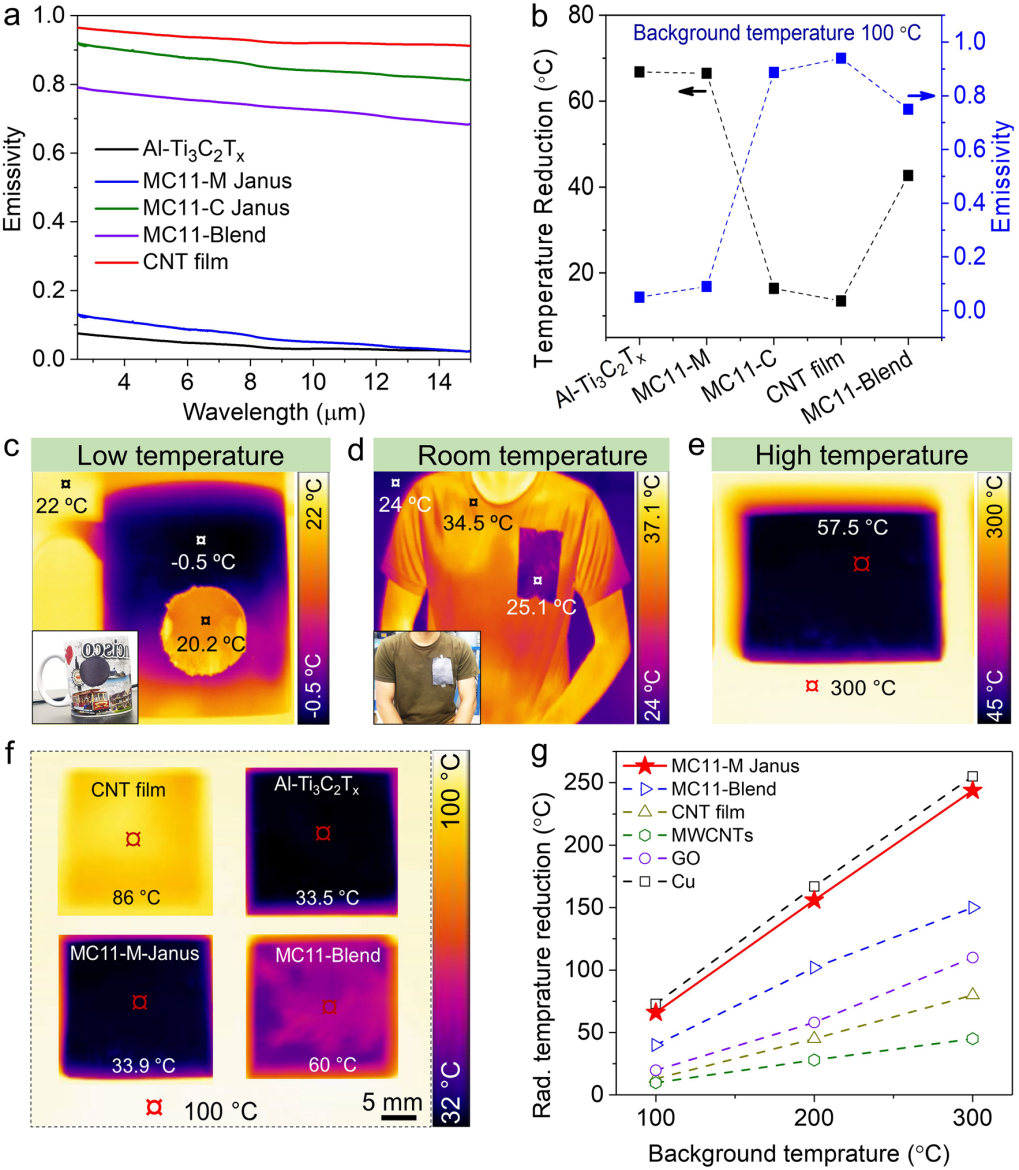
IR emissivity of MXene/CNT Janus film. **a** IR emissivity spectra of the CNT, Al–Ti_3_C_2_T_*x*_ MXene, MC11-C, MC11-M, and MC11-Blend films at room temperature. **b** Relationship between IR emissivity and reduction in the radiation temperature for different samples (background temperature = 100 °C), with the materials exhibiting lower IR emissivity values showing higher reductions in the radiation temperature. **c**–**e** Optical and IR camera images of MC11-M adhered to a ceramic cup filled with ice, a volunteer’s shirt, and onto the hot plate (background temperature 300 °C) respectively, validating its excellent IR-shielding capability at low, ambient, and high temperatures. **f** Radiation temperatures of the CNT, Al–Ti_3_C_2_T_*x*_ MXene, MC11-M, and MC11-Blend films against a background temperature of 100 °C. **g** Reduction in the radiation temperature of MC11-M compared with that of different materials against varying background temperatures

The IR emissivity values of the CNT, Al–Ti_3_C_2_T_x_, MC11-C (CNT side), MC11-M (MXene side), and MC11-Blend films were measured at room temperature in the wavelength range of 2.5–15 μm (Fig. 4a, Table S2). The Al–Ti_3_C_2_T_*x*_ and MC11-M Janus films exhibited remarkably low average IR emissivity values of 0.05 and 0.09, respectively, with the corresponding minimum IR emissivity recorded for both the materials to be 0.02 (Table S2); the obtained values are lower than those of most previously reported MXenes (Fig. S14 and Table S3). The low IR emissivity was likely due to significantly high electrical conductivity of Al–Ti_3_C_2_T_*x*_ (17,500 S cm^−1^), given the inverse relationship between surface IR emissivity and electrical conductivity [83]. Similarly, the Al–Ti_3_C_2_T_*x*_ MXene films exhibited a lower average IR emissivity (0.05) than that of the conv. Ti_3_C_2_T_*x*_ MXene (0.13) (Fig. S15a). Therefore, the Al–Ti_3_C_2_T_*x*_ MXene film showed a considerably larger temperature reduction (33.5 °C) than that of the conv. Ti_3_C_2_T_x_ (38.7 °C) when placed on a hot plate to achieve a surface radiation temperature of 100 °C. This difference was ascribed to the higher electrical conductivity of Al–Ti_3_C_2_T_*x*_ (Fig. S15b, c).

The CNT, MC11-C, and MC11-Blend specimens showed high emissivity values of 0.94, 0.88, and 0.76, respectively, owing to the intrinsically-strong IR absorption capability of the CNT [84]. To comprehensively analyze the thermal camouflage performance of the fabricated samples, a heating stage (hot plate) was employed to simulate a background temperature of 100 °C. Initially, films featuring the CNTs, conv. Ti_3_C_2_T_x_, Al–Ti_3_C_2_T_*x*_, MC Janus specimens having CNTs and MXene as the top-sides (MC-C and MC-M, respectively), and MC11-Blend were positioned on the hot plate being heated at 100 °C. Subsequently, the increase in the radiation temperature over time was recorded using an IR camera for the MC-C and MC-M specimens. The surface radiation temperature of a 10-µm-thick Al–Ti_3_C_2_T_*x*_ film (33.5 °C) was significantly lower than that of the CNTs (83 °C) and MC-C (86 °C) (Fig. S16), due to its higher electrical conductivity. Unlike the MC-C film, the MC-M film exhibited a low surface radiation temperature of 33.9 °C, which was comparable to that of the Al–Ti_3_C_2_T_*x*_ MXene film. An analysis of the relationship between IR emissivity and the radiation temperature reduction for the Al–Ti_3_C_2_T_*x*_ MXene, MC11-M, MC11-C, CNT, and MC11-Blend specimens (Fig. 4b) indicated that the lower the IR emissivity of the materials, the higher the reduction in radiation temperature and the better the thermal camouflage performance of the sample. These results are consistent with those reported previously [83].

The thermal camouflage performance of MC11-M Janus film was evaluated at low, ambient, and high temperatures. At a low temperature, the MC11-M film was attached to a ceramic cup filled with ice. The temperature increased from − 0.5 to 20.2 °C, close to that of air (22 °C) (Fig. 4c). For the room-temperature test, a 10 × 12 cm^2^ MC11-M Janus film was attached to a volunteer’s t-shirt, following which the temperature decreased from 34.5 °C (clothes temperature) to 25.1 °C (sample temperature) (Fig. 4d). For high temperature case, the high-temperature thermal camouflage performance of the MC11-M film was evaluated by putting film onto hot plate with the background temperature of 300 °C. The temperature recorded on the surface of the sample was 57.5 °C (Fig. 4e). Overall, these results underscored the viability of using low-emissivity MC Janus films as IR camouflage materials, potentially permitting the concealment of human bodies from IR detection not only at room temperature but also at extremely low and high temperatures. Furthermore, subjecting the MC11 film to a thermal shock of Δ396 °C for 30 cycles and mechanical bending (6 mm curvature) for 300 cycles did not hamper its thermal camouflage performance (Fig. S17).

The reduction in radiation temperature is a key parameter for evaluating thermal camouflage capability of a material. Notably, the MC11-M Janus film exhibited a significantly higher reduction in the radiation temperature against background temperatures of 100–300 °C than that of several other materials (Fig. 4f, g). This reduction is considerably higher than that of competing nanomaterials including CNTs and graphene, and comparable to that of a Cu metal film, which is deemed the ideal candidate for IR shielding applications because of its exceptionally high electrical conductivity and significantly low IR emissivity.

### Infrared Detection

**Fig. 5:**
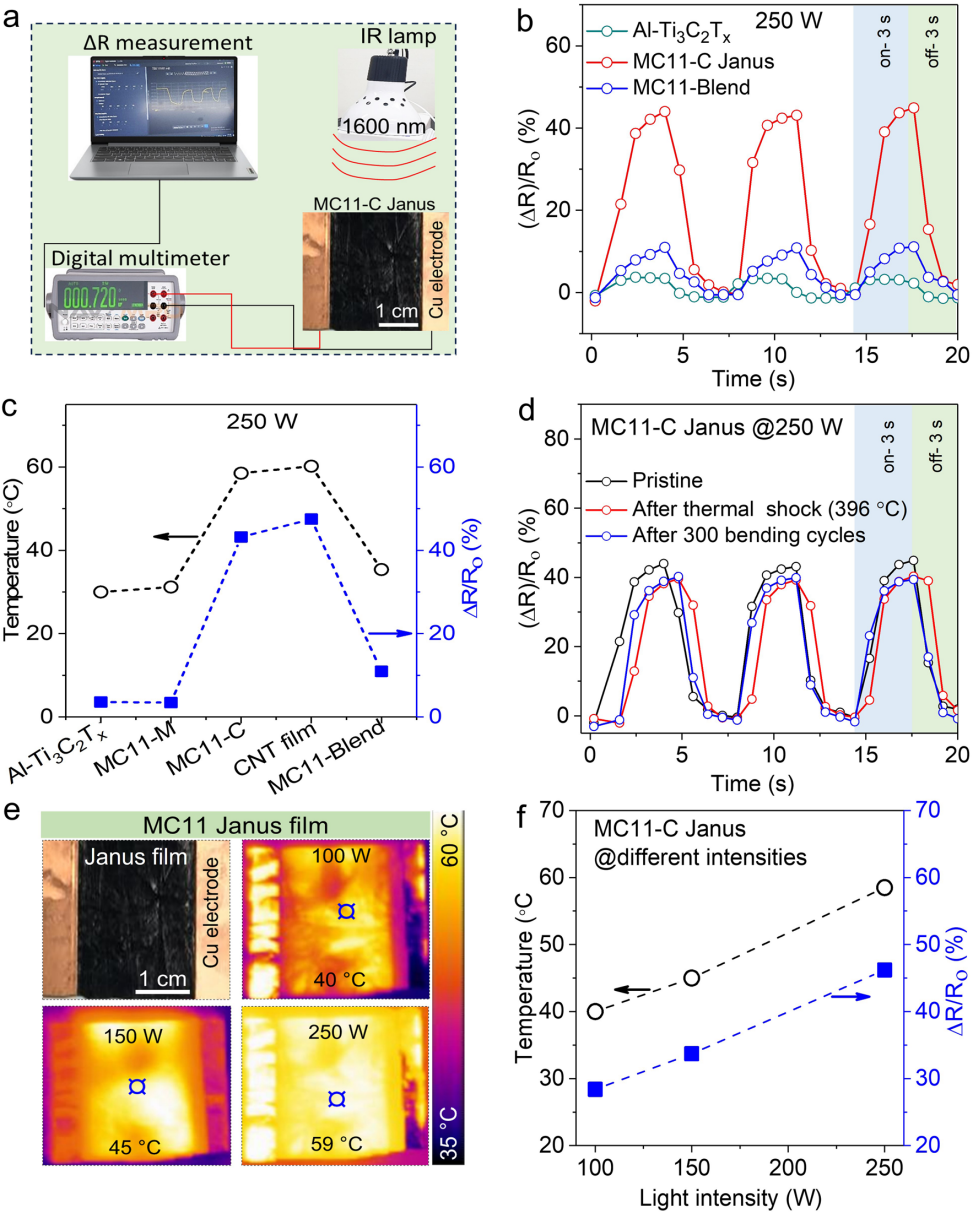
IR detection capability of MXene/CNT Janus film. **a** Schematic of the IR detection setup, featuring the MC11-C sample with Cu electrodes, an IR lamp, and a digital multimeter. **b** Increase in resistance as a function of time for different sample types under 6 s on–off cycling with 250 W IR light. **c** Relationship between changes in temperature and resistance for different samples. **d** Changes in resistance before and after a thermal shock of Δ396 °C (exposure to 200 °C in an oven and to − 196 °C in liquid nitrogen) for 30 cycles, and mechanical bending with 6 mm curvature for 300 cycles. **e** Temperature changes of MC11-C at different light intensities (100, 150, and 250 W). **f** Changes in the temperature and resistance of the MC11-C Janus film at different light intensities (100, 150, and 250 W)

A system featuring a 15-µm-thick MC11-C film placed between two Cu electrodes as the IR radiation detector was constructed to assess its IR detection capability (Fig. 5a). The change in resistance caused by IR radiation is expressed as (*R* − *R*_0_)/*R*_0_ (%), where *R*_0_ is the sample resistance before the IR lamp is switched on, and *R* is the maximum resistance of the sample after IR illumination. The detector sensitivity was quantified as percentage (%) increase in resistance under IR illumination. The Al–Ti_3_C_2_T_*x*_ MXene, MC14, MC12, and MC11 Janus films with both MXene and CNT sides were exposed to 250 W IR radiation (Fig. S18). The CNT and MC-C Janus films exhibited a noteworthy increase in resistance (44%) owing to the excellent IR absorption of the CNT, whereas Al–Ti_3_C_2_T_*x*_ MXene and MC-M films showed a minimal increase in resistance (3.6%) owing to the strong IR-reflecting attributes of the MXene. Furthermore, the increase in resistance of the MC11-blend film fabricated by uniformly mixing the MXene and CNTs was lower (10%) that that of the MC11-C Janus film, but higher than that of the Al–Ti_3_C_2_T_*x*_ MXene film under 6 s on–off cycling (3 s-on, and 3 s-off) (Fig. 5b). The cyclic behavior of the resistance—increase and decrease in the presence and absence of IR illumination, respectively—aligns with the tendencies of a previously reported CNT-based IR detector [85].

The relationship between temperature-increase and change in resistance for the different samples (Fig. 5c) revealed a direct correlation between the two parameters under IR illumination. Notably, the MC11-Blend film showed a smaller change in resistance (10%) than that of the MC11-C Janus film. Uniform mixing helped numerous MXene flakes covering the surface of CNT, thereby increasing the reflection of IR radiation, and consequently reducing the detection sensitivity. The observed increase in resistance can be attributed to the increase in temperature and the presence of photoexcited electrons and holes [86]. Additionally, subjecting the MC11 film to a thermal shock of Δ396 °C for 30 cycles and mechanical bending (6 mm curvature) for 300 cycles barely affected the IR-detecting capability of the MC11-C film (Fig. 5d).

The MC11-C Janus film was illuminated with IR radiation at varying intensities (100, 150, and 250 W) to analyze the effect of the IR intensity on its sensitivity. The acquired IR images (Fig. 5e) and temperature–time spectra (Fig. S19) indicate that under 250 W IR light, the sample exhibited the highest increase in temperature (59 °C), which decreased with decreasing light intensity, *c.a.,* 45 °C for 150 W and 40 °C for 100 W. This increase in temperature can be attributed to the IR-absorbing ability of the sample, which led to an increase in resistance. The thermal energy of the atoms increases with increasing temperature, leading to more frequent collisions between electrons and lattice defects, which impedes electron flow and thereby increases resistance [86]. Additionally, a direct correlation between the increase in temperature and resistance revealed light-intensity-dependent behavior of the change in temperature and resistance (Fig. 5f).

## Conclusion

Electrically-conductive, flexible, robust, and multifunctional MXene/CNT Janus films were fabricated by vacuum-assisted filtration. Incorporating highly crystalline Al–Ti_3_C_2_T_*x*_ MXene on the ozone-treated CNT film facilitated the formation of a Janus film with strong interfacial assembly via hydrogen bonds. The MXene/CNT Janus film exhibited high electrical conductivity as well as excellent tensile strength and mechanical durability, even in harsh environments of extremely high and low temperatures. Consequently, the fabricated Janus film showed an efficient and stable EMI-shielding effectiveness of 72 dB at 15 μm in the X-band, outstanding thermal camouflage performance over a wide temperature range (− 1 to 300 °C) owing to its considerably low IR emissivity (0.09), and high sensitivity (44% increase in resistance under 250 W IR light) towards IR radiation exposure revealing excellent IR detection capability. Therefore, the MXene/CNT Janus film—characterized by an excellent EMI SE value, low IR emissivity, and notable IR detection ability, which were retained in harsh environments—holds strong promise for electronics, military, and aerospace applications.

## Supplementary Information

Below is the link to the electronic supplementary material.

Supplementary file1 (MP4 6513 KB)

Supplementary file2 (MP4 5821 KB)

Supplementary file3 (MP4 8336 KB)

Supplementary file4 (MP4 8507 KB)

Supplementary file5 (MP4 9303 KB)

Supplementary file6 (PDF 1800 KB)
