# Supplementary material for: Multifunctional MXene/Carbon Nanotube Janus Film for Electromagnetic Shielding and Infrared Shielding/Detection in Harsh Environments
Source: Nanomicro Lett. 2024 Jun 14;16:216. doi: 10.1007/s40820-024-01431-3 (PMC11178741; doi:10.1007/s40820-024-01431-3)
Supplement: Supplementary file 6 — Supplementary file6 (PDF 1800 KB) [file 40820_2024_1431_MOESM6_ESM.pdf]

Supporting Information for

Multifunctional MXene/Carbon Nanotube Janus Film for Electromagnetic Shielding and Infrared Shielding/Detection in Harsh Environments

Tufail Hassan[1], Aamir Iqbal[1], Byungkwon Yoo[2], Jun Young Jo[3], Nilufer Cakmakci[2], Shabbir Madad Naqvi[1], Hyerim Kim[1], Sungmin Jung[1], Noushad Hussain[1], Ujala Zafar[1], Soo Yeong Cho[1], Seonghwan Jeong[1], Jaewoo Kim[3], Jung Min Oh[4], Sangwoon Park[4], Youngjin Jeong[2,]*, Chong Min Koo[1,5,]*

[1]School of Advanced Materials Science and Engineering, Sungkyunkwan University, Seobu-ro 2066, Jangan-gu, Suwon-si, Gyeonggi-do, 16419, Republic of Korea

[2]Department of Materials Science and Engineering, Soongsil University, Seoul 06978, Republic of Korea

[3]Institute of Advanced Composite Materials, Korea Institute of Science and Technology, 92 Chudong-ro, Bongdong-eup, Wanju-gun, Jeollabuk-do, 55324, Republic of Korea

[4]R&D Center INNOMXENE Co., Ltd. Daejeon, 34365, Republic of Korea

[5]School of Chemical Engineering, Sungkyunkwan University, Seobu-ro 2066, Jangan-gu, Suwon-si, Gyeonggi-do 16419, Republic of Korea

*Corresponding authors. E-mail: chongminkoo@skku.edu (Chong Min Koo); yjeong@ssu.ac.kr (Youngjin Jeong)

Supplementary Figures and Tables

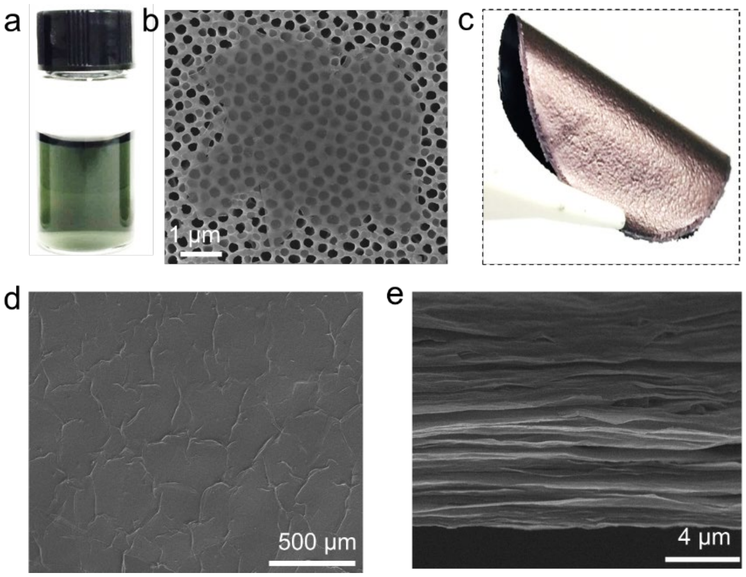

Fig. S1 Overall, morphological, and structural characterization of Al-$Ti_3C_2T_x$ MXene. (**a**) SEM image of a single Al-$Ti_3C_2T_x$ flake. (**b**) Photograph of an aqueous Al-$Ti_3C_2T_x$ MXene dispersion. (**c**) Photograph of a free-standing Al-$Ti_3C_2T_x$ MXene film, signifying its flexibility. (**d**, **e**) SEM images of the surface and cross-section of the Al-$Ti_3C_2T_x$ MXene film

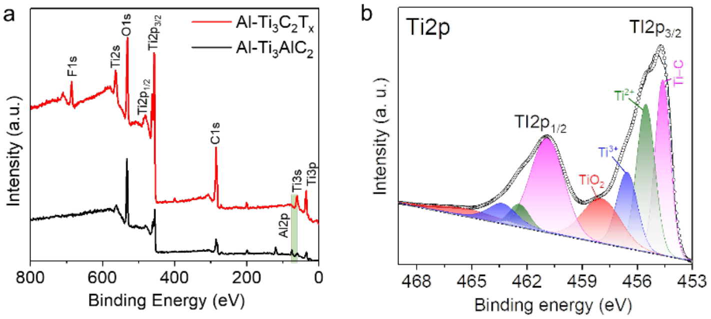

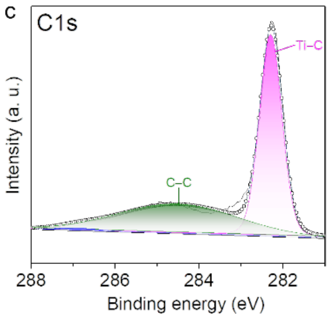

d

Element	Atomic %
Ti	47.5
C	27.7
O	21.3
F	3.5

Fig. S2 (**a**) Broad-scan XPS profiles of the Al-Ti_3AlC_2 MAX phase and Al-$Ti_3C_2T_x$ MXene. (**b, c**) Deconvoluted Ti 2*p* and C 1*s* XPS profiles of the Al-$Ti_3C_2T_x$ MXene. (**d**) Elemental compositions of Al-$Ti_3C_2T_x$ measured from XPS

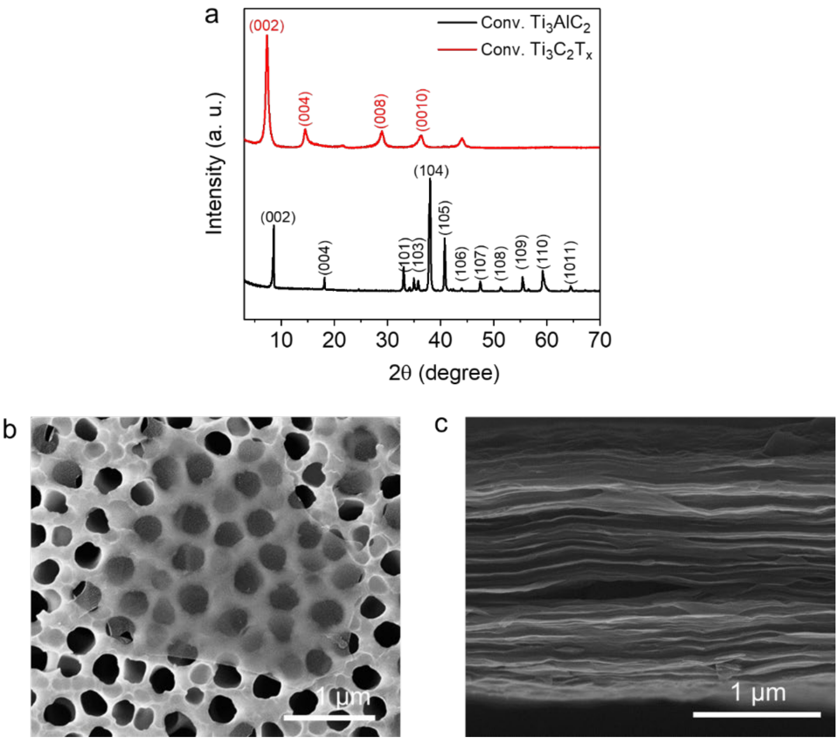

Fig. S3 (**a**) XRD patterns of the conventional Ti_3AlC_2 MAX phase and $Ti_3C_2T_x$ MXene. (**b**) SEM image of a single $Ti_3C_2T_x$ MXene flake. (**c**) Cross-sectional SEM image of the free-standing conventional $Ti_3C_2T_x$ MXene film

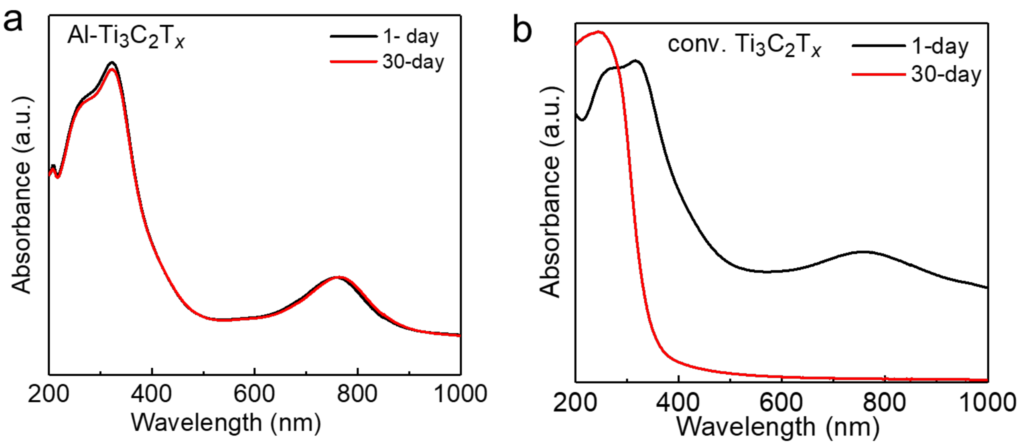

Fig. S4 UV-Visible spectra of (**a**) Al-$Ti_3C_2T_x$ and (**b**) conv. $Ti_3C_2T_x$ aqueous dispersions monitored after 1 and 30 days of storage. There was no observed change in the absorption spectra of Al-$Ti_3C_2T_x$, indicating excellent oxidation stability. In contrast, a significant reduction in absorption was observed for conv. $Ti_3C_2T_x$, indicating poor oxidation stability

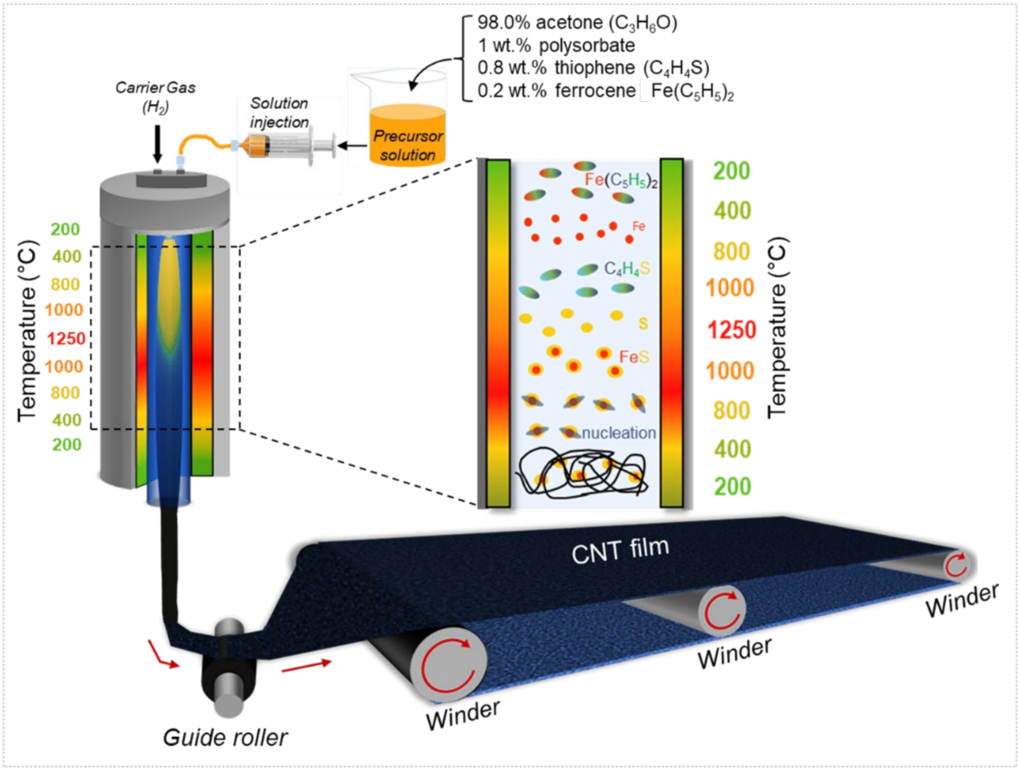

Fig. S5 Schematic illustration of synthesis method of CNTs film via novel modified Chemical Vapor Deposition (CVD) method

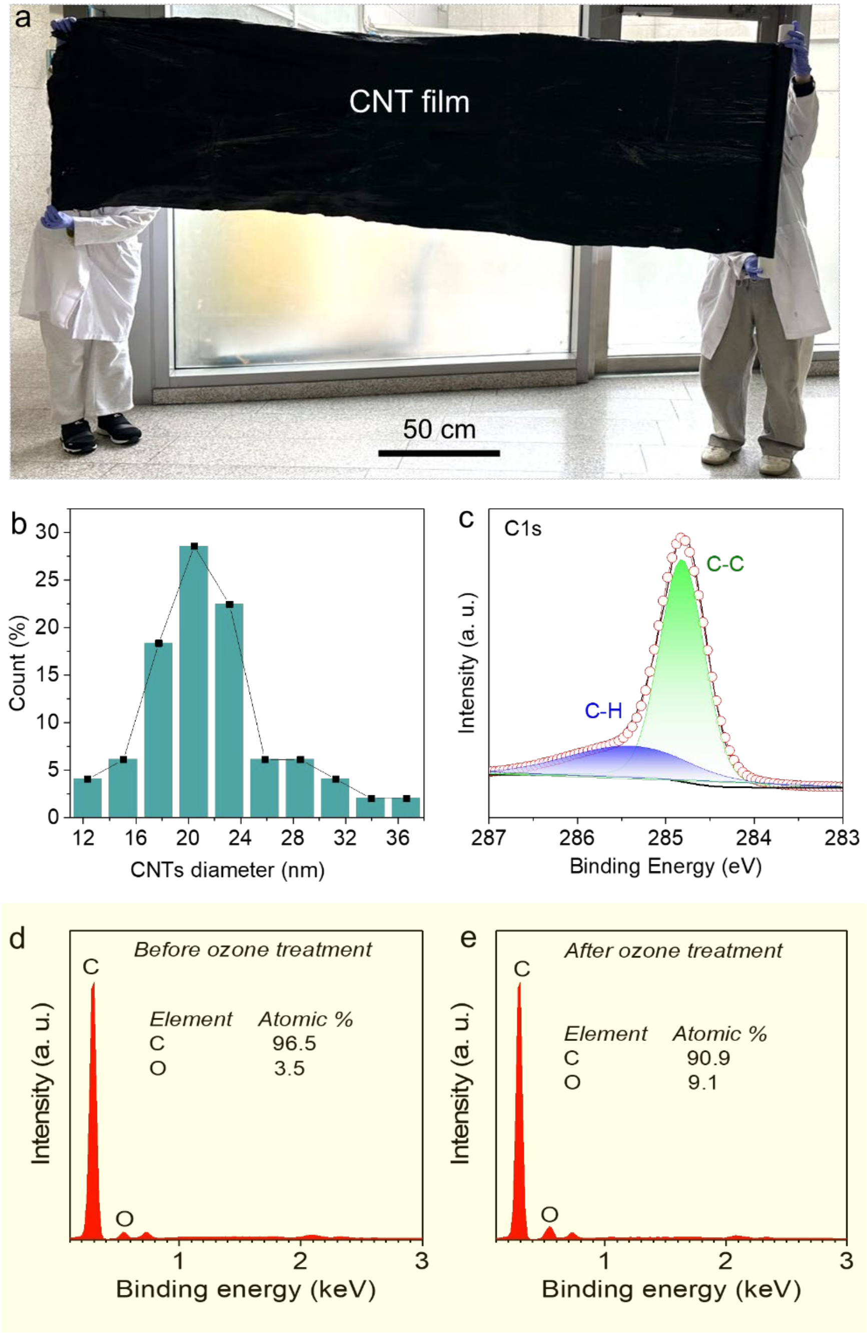

Fig. S6 (**a**) Large area CNTs film (100×300 cm^2) held by a volunteer from each sides (**b**) Average diameter of CNT fibers in CNT film, as calculated from a high-resolution SEM image with Nano-measure software. (**c**) Deconvoluted C1s XPS profile of CNT film. (**d**) EDS elemental composition of the CNT film before ozone treatment. (**e**) EDS elemental composition of the CNT film after ozone treatment, showing an increase in oxygen content

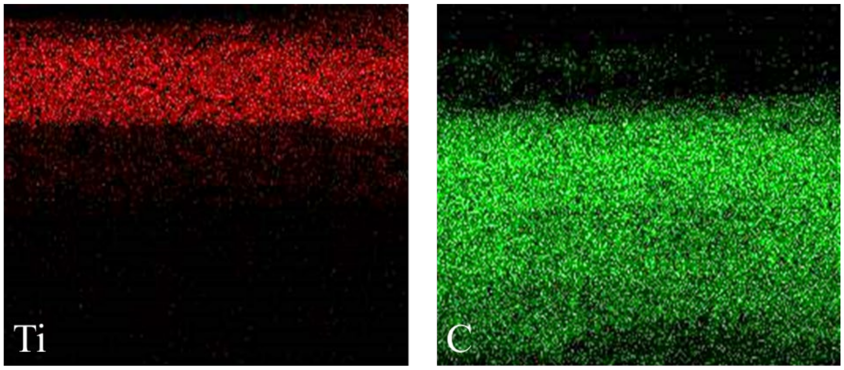

Fig. S7 EDS maps of Ti and C in the cross-section of the MC11-Janus film

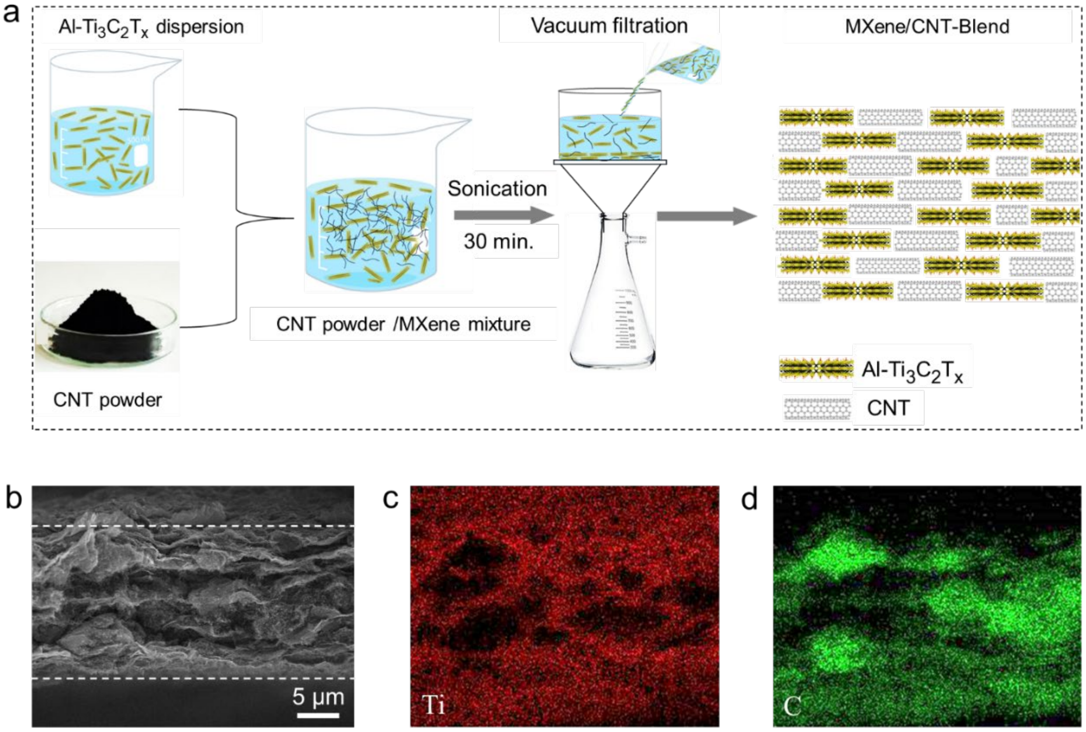

Fig. S8 (**a**) Schematic illustrating the preparation of MXene/CNT-Blend film by solution mixing followed by vacuum-assisted filtration. (**b–d**) Cross-sectional SEM images of the MC11-blend film and the corresponding EDS maps of Ti and C, confirming the uniform distribution of the Al-$Ti_3C_2T_x$ MXene and CNTs throughout the film

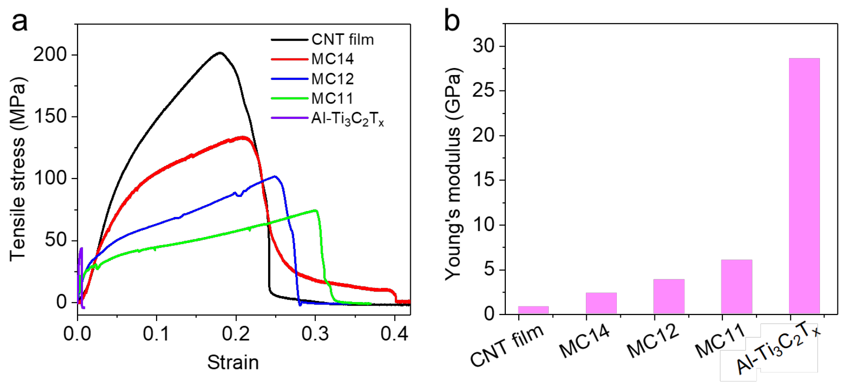

Fig. S9 (**a**) Stress–strain curves, and (**b**) Young's modulus of CNT, MC Janus, and Al-$Ti_3C_2T_x$ films with different compositions, acquired at room temperature

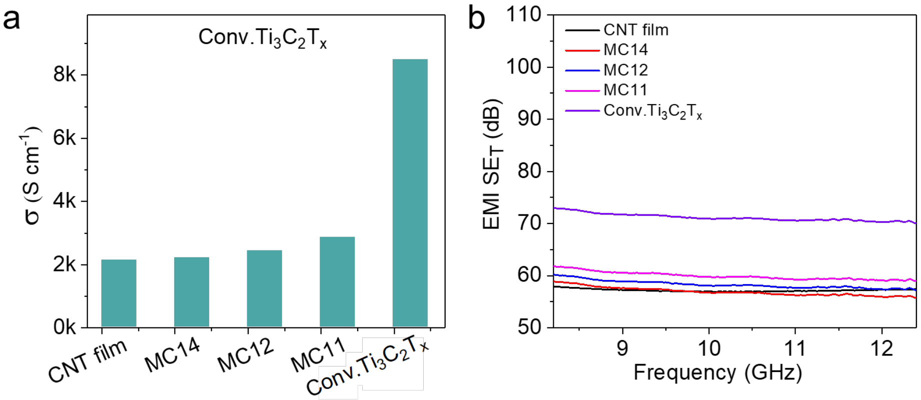

Fig. S10 (**a**) Electrical conductivity, and (**b**) EMI shielding effectiveness (SE_T) values of the CNT (10 μm), Conv.$Ti_3C_2T_x$ MXene (10 μm), and Conv. $Ti_3C_2T_x$ /CNT Janus films including MC14 (11 μm), MC12 (12.5 μm), and MC11 (15 μm)

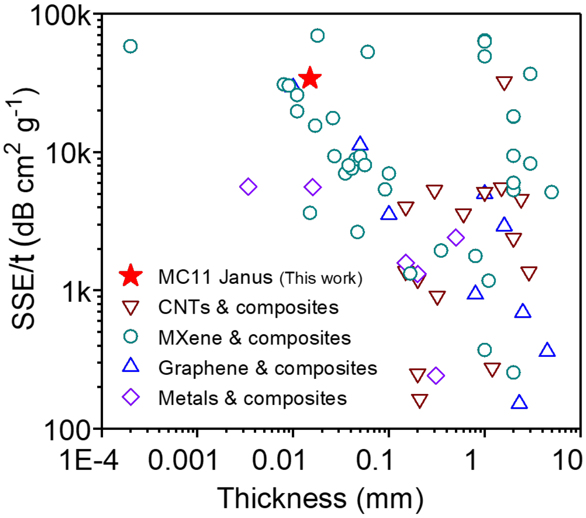

Fig. S11 Specific shielding (SSE/t) values comparison of MC11 Janus films with literature. The SSE/*t* defined as $SE_T/(\rho \bullet t)$) is obtained by dividing the EMI SE_T with the density (ρ) and thickness (t) of material, using as a measure for lightweight and thin-film application capability

Table S1 Specific shielding (SSE/t) of the CM11 Janus film compared with that of several previously reported materials.

Type	**Materials**	**Thickness (cm)**	**Density (g cm^{-3})**	**EMI SE (dB)**	**SSE/t (dB cm^{2} g^{-1})**	**Refs.**
Graphene and composites	Aligned rGO/Epoxy	0.01	1.07	38	3530.0	[S1]
	rGO/PS	0.25	0.26	45.1	692.0	[S2]
	rGO/PDMS	0.1	0.06	30	5000.0	[S3]
	rGO/PEI	0.23	0.29	10	152.2	[S4]
	Graphene film	0.001	1.49	43.8	29396.0	[S5]
	rGO/PMMA	0.4	0.79	19	60.0	[S6]
	CNWs/G-PDMS	0.16	0.097	36	2919.6	[S7]
	Graphene/PMMA	0.34	1.19	30	74.2	[S8]
	Graphene pallet	0.005	1.07	60	11215.0	[S9]
	rGO/PI	0.08	0.28	21	937.5	[S10]
	GF@PDMS	0.45	0.22	35.8	361.6	[S11]
CNTs and composites	CNT-sponge	0.24	0.02	22	4583.0	[S12]
	MWCNT/WPU	0.1	0.04	21.1	5140.0	[S13]
	SWCNT/PS	0.12	0.56	18.5	275.0	[S14]
	CNF mat	0.29	0.134	52.2	1361.6	[S15]
	MWCNT/GF/PDMS	0.15	0.09	75	5556.0	[S16]
	MWCNT-based composite paper	0.06	0.26	56	3583.0	[S17]
	MWCNT/CNF	0.015	0.77	46.4	4017.3	[S18]
	MWCNT/WPU	0.032	1.2	35	911.5	[S19]
	MWCNT PEO/cellulose	0.15	1.7	35	1372.5	[S20]
	Fe_3O_4/MWCNT/phenolic carbon foam	0.2	0.13	62	2385.0	[S21]
	Wood-derived carbon grids	0.03	0.28	44.5	5297.6	[S22]
	CNT/MLGEP	0.16	0.0089	47	32375.0	[S23]
	MWCNT/PC	0.021	1.13	39	164.0	[S24]
	MWCNT/PPCP	0.02	0.94	47	250.0	[S25]
	Carbon foam	0.02	0.17	40	1200.0	[S26]
Metals and composites	SF/PP foams	0.31	0.64	48	241.9	[S27]
	CuNi-CNT	0.15	0.23	54.6	1580.0	[S28]
	AgNW/PI	0.5	0.029	35	2416.0	[S29]
	GCC film	0.0034	2.64	51	5632.1	[S30]
	AgNW/cellulose papers	0.016	0.53	48.6	5585.0	[S31]
	cMF-Au-GIO/PDMS	0.2	0.12	30.5	1314.7	[S32]
MXene and Composites	MXene/CNF	0.0167	1.129	25	1326.0	[S33]
		0.0047	2.09	26	2647.0	
	MXene Foam	0.006	0.22	70	53030.0	[S34]
	$Ti_3C_2T_x$/SA	0.0008	2.31	57	30830.0	[S35]
	$Ti_3C_2T_x$ thin film	0.0011	2.39	68	25863.0	
	$Ti_3C_2T_x$ /CNF	0.0038	1.26	37.7	7874.0	[S36]
	rGO/ $Ti_3C_2T_x$	0.2	0.3	56.4	9400.0	[S37]
	$Ti_3C_2T_x$/PEDOT:PSS	0.0015	1.65	9	3636.0	[S38]
		0.0011	1.94	42.1	19728.0	
	$Ti_3C_2T_x$/PS	0.2	1.08	62	255.2	[S39]
	$Ti_3C_2T_x$/wax	0.1	2.05	76.1	371.0	[S40]

	$Ti_3C_2T_x$/PVA	0.5	0.011	28	5136.0	[S41]
	$Ti_3C_2T_x$/CNF	0.0035	1.63	40	7011.0	[S42]
	ANF- $Ti_3C_2T_x$/ AgNW (10 wt%)	0.0041	1.14	35.5	7595.2	[S43]
	ANF- $Ti_3C_2T_x$/ AgNW (20 wt%)	0.0045	1.2	48.1	8907.0	
	ANF-MXene/ AgNW (40 wt%)	0.005	1.23	57.3	9317.1	
	ANF- $Ti_3C_2T_x$/ AgNW (60 wt%)	0.0056	1.42	64.1	8060.9	
	ANF- $Ti_3C_2T_x$/ AgNW (80 wt%)	0.0091	1.63	79.8	5379.9	
	$Ti_3C_2T_x$/Wax	0.08	2.03	70	1776.0	[S44]
	$Ti_3C_2T_x$ /HEC	0.01	0.34	24	7000.0	[S45]
	$Ti_3C_2T_x$ /ANF	0.0017	1.25	33	15529.0	[S46]
	$Ti_3C_2T_x$ /TiO_2/rGO	0.0009	1.01	28	30293.0	[S47]
	$Ti_3C_2T_x$ /rGO/PVDF	0.035	0.79	54	1944.0	[S48]
	$Ti_3C_2T_x$ /Ni/PVDF	0.11	1.65	19.5	1177.0	[S49]
	$Ti_3C_2T_x$ /CNT	0.00002	2.49	2.9	58187.0	[S50]
	$Ti_3C_2T_x$ /PVA	0.0027	1.74	44.4	9343.0	[S51]
	$Ti_3C_2T_x$	0.1	0.109	70.6	64182.0	[S52]
	Ti_3CNT_x	0.1	0.11	69.2	62909.0	
	$Ti_3C_2T_x$	0.1	0.109	54.1	49182.0	
	$Ti_3C_2T_x$ /rGO	0.3	0.046	50.7	36737.0	[S53]
	$Ti_3C_2T_x$ /AgNW	0.2	0.49	52.6	5313.0	[S54]
	$Ti_3C_2T_x$ /SA/PDMS	0.2	0.02	72	18000.0	[S55]
	$Ti_3C_2T_x$	0.2	0.206	75	18116.0	[S56]
	$Ti_3C_2T_x$ /CNT	0.3	0.42	104	8253.0	[S57]
	$Ti_3C_2T_x$ /CA	0.0026	1.18	54.3	17586.0	[S58]
**Present study**	**MC11 Janus**	**0.0015**	**1.41151**	**72**	**34042.5**	
	**Al-$Ti_3C_2T_x$**	**0.0010**	**3.21**	**88**	**27414.33**	
	**MC11-Blend**	**0.0015**	**1.51**	**60**	**26490.1**	

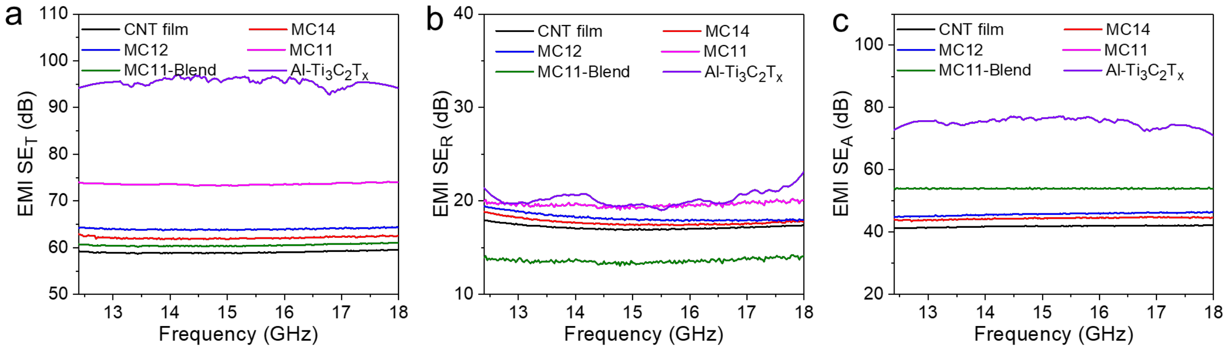

Fig. S12 (**a**) SE_T (**b**) SE_R, and (**c**) SE_A value of the CNT film (10 μm), MC14 (11 μm), MC12 (12.5 μm), MC11 (15 μm), MC11-Blend (15 μm), and Al-$Ti_3C_2T_x$ MXene (10 μm) film in Ku band, covering a frequency range from 12.4-18 GHz

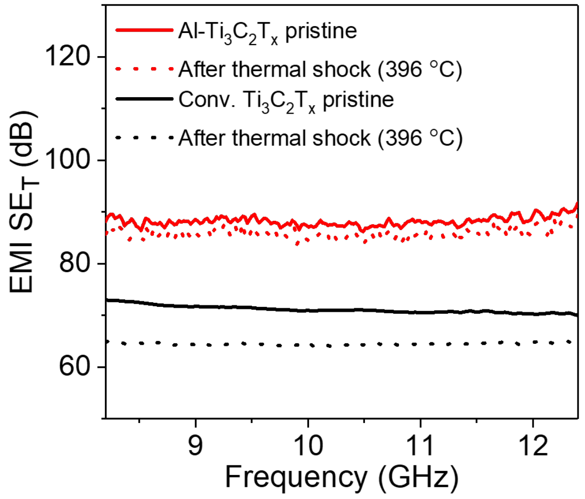

Fig. S13 SE_T values of conventional $Ti_3C_2T_x$ and Al-$Ti_3C_2T_x$ before and after undergoing thermal shock (396 °C) for 30 cycles

Table S2 IR emissivity values of fabricated films

Sample	Minimum emissivity	Average emissivity
Al-$Ti_3C_2T_x$	0.02	0.05
MC11-M Janus	**0.02**	**0.09**
MC11-C Janus	0.81	0.887
CNT film	0.911	0.94
MC11-Blend	0.68	0.76
Conv. $Ti_3C_2T_x$	0.06	0.13

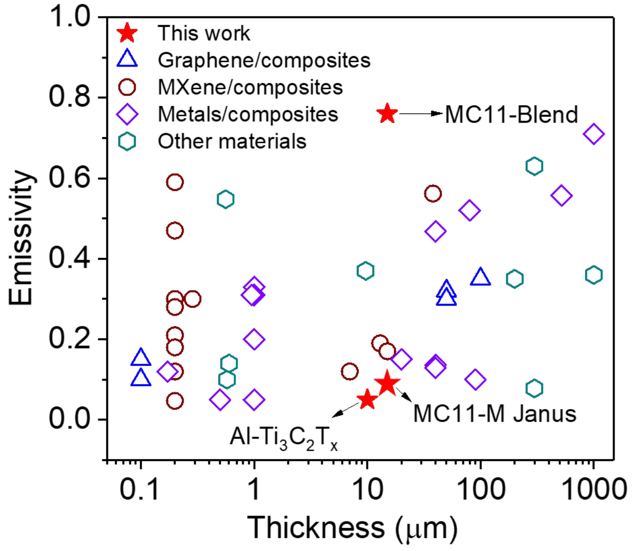

Fig. S14 IR emissivity of the MC11-M Janus film compared with those of MC11-Blend, the Al-$Ti_3C_2T_x$, and other materials

Table S3 IR emissivity of the MC11-M Janus film compared with that of several previously reported materials.

	Materials	Sample type/Preparation method	Wavelength range (μm)	Thickness (μm)	Emissivity	Ref.
Graphene and composites	Graphene	Flexible film/Vacuum filtration	2-18	-	0.32	[S59]
	Graphene Oxide	Flexible film/Vacuum filtration	2-18	-	0.85	
	CNTs	-	2-18		0.76	
	Multilayer graphene	-		50	0.32	[S60]
	Graphene/PE + IL/Au	Flexible Film/CVD	2-25	50	0.3	
	Graphene/Fabric + IL/Au	Flexible Film/CVD	8-13	100	0.35	[S61]
	Graphene/Celgard + IL/Au	Flexible Film/CVD	7.5-13	0.1	0.1	[S62]
	Graphene/Celgard + IL/ graphene	Flexible Film/CVD	7.5-14	0.1	0.1	
	Graphene/Celgard + IL/Cu	Flexible Film/CVD	7.5-15	0.1	0.1	
	graphene/PES + IL/Au	Flexible Film/ CVD	5-20	-	0.65	[S63]
MXene and composites	MXene− TOCNF	Flexible Film/Blade coating	7-14	38	0.562	[S64]
	$Ti_3C_2T_x$	Flexible Film/Vacuum filtration	7-14	13	0.19	[S65]
				29		
				45		
	$Ti_3C_2T_x$	Flexible Film/Vacuum filtration	2-18	-	0.14	[S59]
	$Ti_3C_2T_x$ film	Flexible Film/ Vacuum filtration	7-14	-	0.09	[S66]
	$Ti_3C_2T_x$/Graphene layer-by-layer film	Flexible Film/Fabric/Vacuum filtration	7-14	7	0.12	
	$Ti_3C_2T_x$	Flexible Film/Fabric/Vacuum filtration	3-16.7	15	0.17	[S67]
	$Ti_3C_2T_x$/Elastomer Bilayer Structure	Flexible Film/HF etching	7-17	0.286	0.286	[S68]
	Nb_2CT_x	/Spray coated thin film	0-25	0.2	0.59	[S69]
	$Nb_4C_3T_x$	/Spray coated thin film	0-25	0.2	0.47	
	$Mo_2Ti_2C_3T_x$	/Spray coated thin film	0-25	0.2	0.3	
	V_2CT_x	/Spray coated thin film	0-25	0.2	0.28	
	$V_4C_3T_x$	/Spray coated thin film	0-25	0.2	0.21	
	Ti_2CT_x	/Spray coated thin film	0-25	0.2	0.18	
	Ti_3CNT_x	/Spray coated thin film	0-25	0.2	0.12	
	$Ti_3C_2T_x$	/Spray coated thin film	0-25	0.2	0.047	

Metals and composites	Ag	-	2-18	-	0.049	[S59]
	Cu	-	2-18	-	0.11	
	Al	-	2-18	-	0.072	
	Stainless steel	-	2-18	-	0.14	
	30%Al/PR	/Spray Coating technique/Rigid	8-14	-	0.24	[S70]
	Al@SiO_2/EPDM	/Sol-gel, spray technique/Rigid	8-14	40	0.468	[S71]
	Al–SiO_2	Rigid film/Vacuum magnetron sputtering	8-14	0.172	0.12	[S72]
	Polyethylene wax/Al	Coating/Flux-capping method/ Rigid	8-14	80	0.52	[S73]
	20%Cu/EPDM-g-MAH	/Spray Coating/Rigid	8-14	20	0.15	[S74]
	50%Cu/PU	/Spray Coating/Rigid	8-14	90	0.1	[S75]
	50%Ag/PU	/Knife coating process/Rigid	8-14	40	0.136	[S76]
	(Ball-milled Ag–Cu)/PU	/Knife coating process/Rigid	8-14	40	0.129	
	SiO_2/Ag/TiO_2	Composite /Chemical deposition/Rigid	8-14	520	0.557	[S77]
	Pt–Ag	/Radio-frequency magnetron sputtering/Rigid	8-14	1000	0.71	[S78]
	Ag/Ge	Flexible film/Electron-beam evaporation	8-14	1	0.31	[S79]
	Cu/PET/ZnSe	Film/Copper electrodeposition/Rigid	8-12	1	0.2	[S80]
	Pt film	-		0.5	0.05	[S81]
	Si/GST/Au	-	3-14	1	0.33	[S82]
	Au	-		1	0.05	[S83]
Other materials	TiN_x film	-		0.577	0.1	[S84]
	Al/ATO	Composite/Coprecipitation	8-14		0.708	[S85]
	Ge/ZnS/SiO_2 aerogel	Flexible film/Electron-beam evaporation	8-14	300	0.078	[S86]
	SiO_2/TiO_2/Polyacetylene multilayered nanospheres	Composite/Chemical reaction/Rigid	8-14	0.56	0.548	[S87]
	W@VO_2	Flexible film/ Pulsed laser deposition	5-17	200.09	0.35	[S88]
	VO_2	/Hydrothermal	8-14	1000	0.36	[S89]

	Ge/TiO_2	/Electron beam coating/Rigid photonic crystal	8-14	-	0.202	[S90]
	Si	-	-	-	0.7	[S59]
	Ge	-	-	-	0.7	
	Leather/SiO_2			300	0.63	[S91]
	Co_3O_4	-	-		0.71	[S92]
	ZrB_2	-	-	2000	0.09	[S93]
	TiB2	-	-	2000	0.15	
	CdTe	-	-	9.7	0.37	[S94]
	Au/ZnS/Au	-	2-18	0.6	0.14	[S95]
	MZT	-	-	2100	-	[S96]
**Present study**	**MC11-M Janus**	**Vacuum filtration**	**2-14**	**15**	**0.09**	
	**Conv.$Ti_3C_2T_x$**	**Vacuum filtration**	**2-14**	**10**	**0.13**	
	**Al-$Ti_3C_2T_x$**	**Vacuum filtration**	**2-14**	**10**	**0.05**	
	**MC11-Blend**	**Vacuum filtration**	**2-14**	**15**	**0.76**	

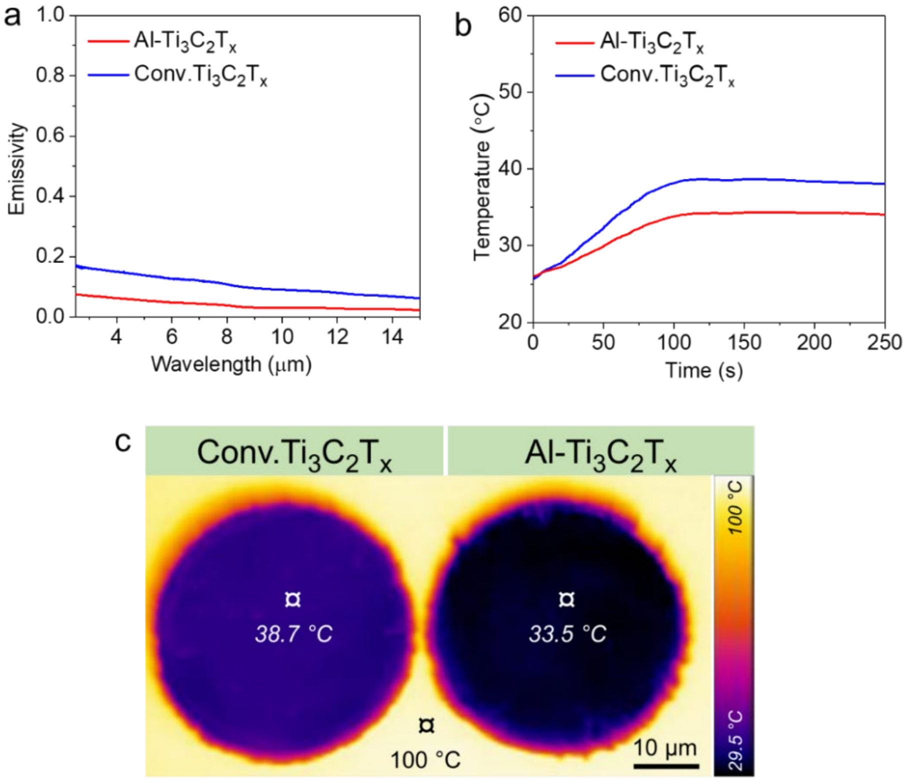

Fig. S15 (**a**) IR emissivity data. (**b, c**) Time-dependent temperature changes of the Al-$Ti_3C_2T_x$ and conventional $Ti_3C_2T_x$ films against a background temperature of 100 °C, and the corresponding photographs

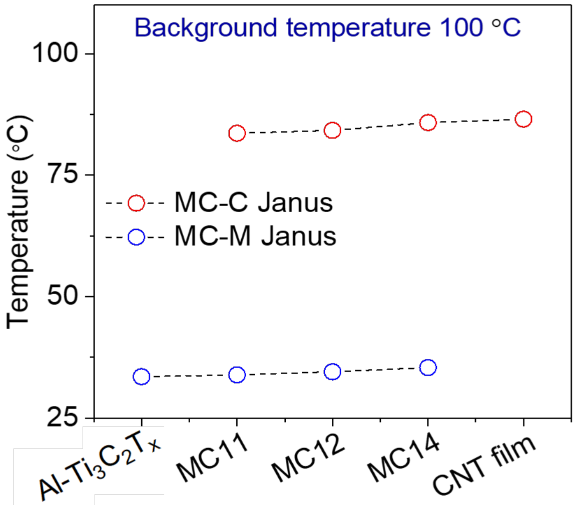

Fig. S16 Thermal camouflage performances of the CNT, conventional $Ti_3C_2T_x$ and Al-$Ti_3C_2T_x$ MXene, and MC Janus-C (CNTs side) and MC11 Janus-M (MXene side) films

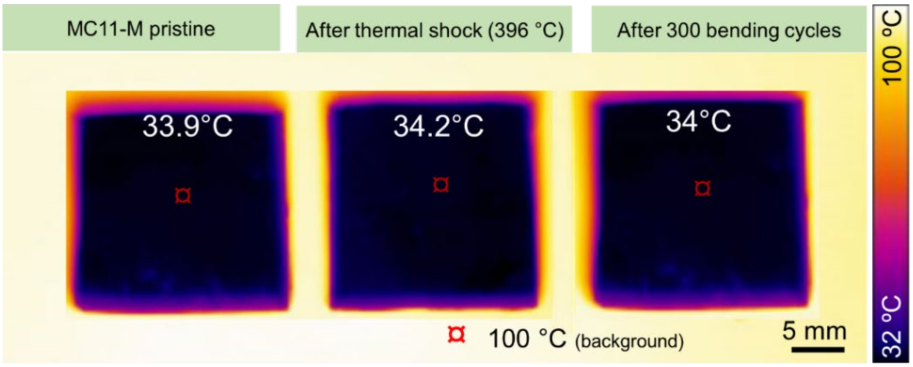

Fig. S17 Thermal camouflage performance retention of the MC11-M film before and after undergoing thermal shock (396 °C) for 30 cycles, and after 300 bending cycles at a radius of 6 mm

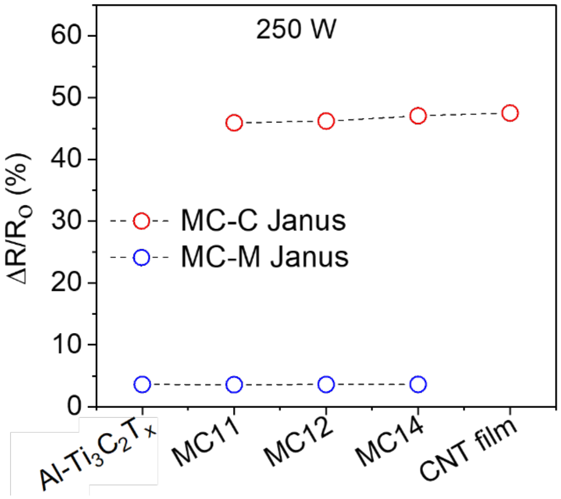

Fig. S18 IR-detecting capabilities of the CNT, Al-MXene, and MC Janus-C (CNT side) and MC11 Janus-M (MXene side) films

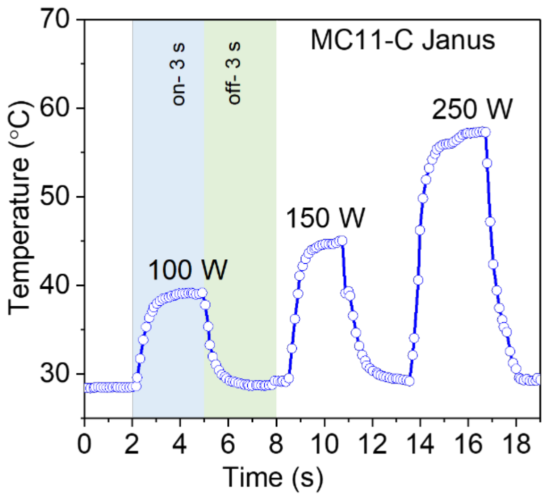

Fig. S19 Temperature change of MC11-C at different light intensities of 100W, 150W, and 250W in a 6-sec cycle (on-3 sec, and off-3 sec)

Supplementary References

[S1] N. Yousefi, Sun X., Lin X., Shen X., Jia J. et al., Highly aligned graphene/polymer nanocomposites with excellent dielectric properties for high-performance electromagnetic interference shielding. Adv. Mater. **26**, 5480–5487 (2014). https://doi.org/10.1002/adma.201305293

[S2] Yan D.-X., Pang H., Li B., R. Vajtai, Xu L. et al., Structured reduced graphene oxide/polymer composites for ultra-efficient electromagnetic interference shielding. Adv. Funct. Mater. **25**, 559–566 (2015). https://doi.org/10.1002/adfm.201403809

[S3] Z. Chen, C. Xu, C. Ma, W. Ren, H.-M. Cheng Lightweight and flexible graphene foam composites for high-performance electromagnetic interference shielding. Adv. Mater. **25**, 1296–1300 (2013). https://doi.org/10.1002/adma.201204196

[S4] J. Ling, W. Zhai, W. Feng, B. Shen, J. Zhang et al., Facile preparation of lightweight microcellular polyetherimide/graphene composite foams for electromagnetic interference shielding. ACS Appl. Mater. Interfaces **5**, 2677–2684 (2013). https://doi.org/10.1021/am303289m

[S5] Q. Wei, S. Pei, X. Qian, H. Liu, Z. Liu et al., Superhigh electromagnetic interference shielding of ultrathin aligned pristine graphene nanosheets film. Adv. Mater. **32**, e1907411 (2020). https://doi.org/10.1002/adma.201907411

[S6] H.-B. Zhang, Q. Yan, W.-G. Zheng, Z. He, Z.-Z. Yu Tough graphene–polymer microcellular foams for electromagnetic interference shielding. ACS Appl. Mater. Interfaces **3**, 918–924 (2011). https://doi.org/10.1021/am200021v

[S7] L. Kong, X. Yin, M. Han, X. Yuan, Z. Hou et al., Macroscopic bioinspired graphene sponge modified with in-situ grown carbon nanowires and its electromagnetic properties. Carbon **111**, 94-102 (2017). https://doi.org/10.1016/j.carbon.2016.09.066

[S8] H.B. Zhang, W.G. Zheng, Q. Yan, Z.G. Jiang, Z.Z. Yu, The effect of surface chemistry of graphene on rheological and electrical properties of polymethylmethacrylate composites. Carbon **50**, 5117–5125 (2012). https://doi.org/10.1016/j.carbon.2012.06.052

[S9] L. Zhang, N. T. Alvarez, M. Zhang, M. Haase, R. Malik et al., Preparation and characterization of graphene paper for electromagnetic interference shielding. Carbon. **82**, 353-359 (2015). https://doi.org/10.1016/j.carbon.2014.10.080

[S10] Y. Li, X. Pei, B. Shen, W. Zhai, L. Zhang, W. Zheng, Polyimide/graphene composite foam sheets with ultrahigh thermostability for electromagnetic interference shielding. RSC Adv. **5**(31), 24342-24351 (2015). https://doi.org/10.1039/C4RA16421K

[S11] Li H., Jing L., Z.L. Ngoh, R.Y. Tay, Lin J. et al., Engineering of high-density thin-layer graphite foam-based composite architectures with superior compressibility and excellent electromagnetic interference shielding performance. ACS Appl. Mater. Interfaces **10**, 41707–41716 (2018). https://doi.org/10.1021/acsami.8b15240

[S12] M. Crespo, M. González, A. L. Elías, L. Pulickal Rajukumar, J. Baselga, M. Terrones, J. Pozuelo. Ultra-light carbon nanotube sponge as an efficient electromagnetic shielding material in the GHz range. RRL **8**(8), 698-704 (2014). https://doi.org/10.1016/j.carbon.2016.10.031

[S13] Z. Zeng, H. Jin, M. Chen, W. Li, L. Zhou et al., Lightweight and anisotropic porous MWCNT/WPU composites for ultrahigh performance electromagnetic interference shielding. Adv. Funct. Mater. **26**, 303–310 (2016). https://doi.org/10.1002/adfm.201503579

[S14] Y. Yang, M.C. Gupta, K.L. Dudley, R.W. Lawrence Novel carbon nanotube-polystyrene foam composites for electromagnetic interference shielding. Nano Lett. **5**, 2131–2134 (2005). https://doi.org/10.1021/nl051375r

[S15] X. Hong, D. Chung. Carbon nanofiber mats for electromagnetic interference shielding. Carbon **111**, 529-537 (2017). https://doi.org/10.1016/j.carbon.2016.10.031

[S16] Sun X., Liu X., Shen X., Wu Y., Wang Z. et al., Graphene foam/carbon nanotube/poly(dimethyl siloxane) composites for exceptional microwave shielding. Compos. Part A Appl. Sci. Manuf. **85**, 199–206 (2016). https://doi.org/10.1016/j.compositesa.2016.03.009

[S17] A. Chaudhary, S. Kumari, R. Kumar, S. Teotia, B.P. Singh et al., Lightweight and easily foldable MCMB-MWCNTs composite paper with exceptional electromagnetic interference shielding. ACS Appl. Mater. Interfaces **8**, 10600–10608 (2016). https://doi.org/10.1021/acsami.5b12334

[S18] H. Zhang, X. Sun, Z. Heng, Y. Chen, H. Zou et al., Robust and flexible cellulose nanofiber/multiwalled carbon nanotube film for high-performance electromagnetic interference shielding. Ind. Eng. Chem. Res. **57**, 17152–17160 (2018). https://doi.org/10.1021/acs.iecr.8b04573

[S19] Z. Zeng, M. Chen, H. Jin, W. Li, X. Xue et al., Thin and flexible multi-walled carbon nanotube/waterborne polyurethane composites with high-performance

electromagnetic interference shielding. Carbon **96**, 768–777 (2016). https://doi.org/10.1016/j.carbon.2015.10.004

[S20] L.-Q. Zhang, B. Yang, J. Teng, J. Lei, D.-X. Yan et al., Tunable electromagnetic interference shielding effectiveness via multilayer assembly of regenerated cellulose as a supporting substrate and carbon nanotubes/polymer as a functional layer. J. Mater. Chem. C **5**, 3130–3138 (2017). https://doi.org/10.1039/c6tc05516h

[S21] Q. Li, L. Chen, J. Ding, J. Zhang, X. Li et al., Open-cell phenolic carbon foam and electromagnetic interference shielding properties. Carbon **104**, 90-105 (2016). https://doi.org/10.1016/j.carbon.2016.03.055

[S22] Z. Chen, D. Yi, B. Shen, L. Zhang, X. Ma et al., Semi-transparent biomass-derived macroscopic carbon grids for efficient and tunable electromagnetic shielding. Carbon **139**, 271–278 (2018). https://doi.org/10.1016/j.carbon.2018.06.070

[S23] Q. Song, F. Ye, X. Yin, W. Li, H. Li et al., Carbon nanotube–multilayered graphene edge plane core–shell hybrid foams for ultrahigh-performance electromagnetic-interference shielding. Adv. Mater. **29**, 1701583 (2017). https://doi.org/10.1002/adma.201701583

[S24] S. Pande, A. Chaudhary, D. Patel, B.P. Singh, R.B. Mathur Mechanical and electrical properties of multiwall carbon nanotube/polycarbonate composites for electrostatic discharge and electromagnetic interference shielding applications. RSC Adv. **4**, 13839–13849 (2014). https://doi.org/10.1039/C3RA47387B

[S25] P. Verma, P. Saini, R. S. Malik, V. Choudhary, Excellent electromagnetic interference shielding and mechanical properties of high loading carbon-nanotubes/polymer composites designed using melt recirculation equipped twin-screw extruder. Carbon **89**, 308-317 (2015). https://doi.org/10.1016/j.carbon.2015.03.063

[S26] F. Moglie, D. Micheli, S. Laurenzi, M. Marchetti, V. Mariani Primiani Electromagnetic shielding performance of carbon foams. Carbon **50**, 1972–1980 (2012). https://doi.org/10.1016/j.carbon.2011.12.053

[S27] Ameli, M. Nofar, Wang S., C.B. Park Lightweight polypropylene/stainless-steel fiber composite foams with low percolation for efficient electromagnetic interference shielding. ACS Appl. Mater. Interfaces **6**, 11091–11100 (2014). https://doi.org/10.1021/am500445g

[S28] K. Ji, H. Zhao, J. Zhang, J. Chen, Z. Dai, Fabrication and electromagnetic interference shielding performance of open-cell foam of a Cu–Ni alloy integrated with cnts. Appl. Surface Sci. **311**, 351-356 (2014). https://doi.org/10.1016/j.apsusc.2014.05.067

[S29] J. Ma, K. Wang, M. Zhan A comparative study of structure and electromagnetic interference shielding performance for silver nanostructure hybrid polyimide foams. RSC Adv. **5**, 65283–65296 (2015). https://doi.org/10.1039/c5ra09507g

[S30] M. Yang, Q. Wei, J. Li, Y. Wang, H. Guo et al., Flexible composite carbon films prepared by a pancake-making method for electromagnetic interference shielding. Adv. Mater. Interfaces **7**, 1901815 (2020). https://doi.org/10.1002/admi.201901815

[S31] T.W. Lee, S.E. Lee, Y.G. Jeong Highly effective electromagnetic interference shielding materials based on silver nanowire/cellulose papers. ACS Appl. Mater. Interfaces **8**, 13123–13132 (2016). https://doi.org/10.1021/acsami.6b02218

[S32] Sun Y., Luo S., Sun H., Zeng W., Ling C. et al., Engineering closed-cell structure in lightweight and flexible carbon foam composite for high-efficient electromagnetic interference shielding. Carbon **136**, 299–308 (2018). https://doi.org/10.1016/j.carbon.2018.04.084

[S33] W.-T. Cao, F.-F. Chen, Y.-J. Zhu, Y.-G. Zhang, Y.-Y. Jiang et al., Binary strengthening and toughening of MXene/cellulose nanofiber composite paper with nacre-inspired structure and superior electromagnetic interference shielding properties. ACS Nano **12**, 4583–4593 (2018). https://doi.org/10.1021/acsnano.8b00997

[S34] J. Liu, H.-B. Zhang, R. Sun, Y. Liu, Z. Liu et al., Hydrophobic, flexible, and lightweight MXene foams for high-performance electromagnetic-interference shielding. Adv. Mater. **29**, 1702367 (2017). https://doi.org/10.1002/adma.201702367

[S35] F. Shahzad, M. Alhabeb, C.B. Hatter, B. Anasori, S. Man Hong et al., Electromagnetic interference shielding with 2D transition metal carbides (MXenes). Science **353**, 1137–1140 (2016). https://doi.org/10.1126/science.aag2421

[S36] W. Cao, C. Ma, S. Tan, M. Ma, P. Wan et al., Ultrathin and flexible CNTs/MXene/cellulose nanofibrils composite paper for electromagnetic interference shielding. Nano-Micro Lett. **11**, 72 (2019). https://doi.org/10.1007/s40820-019-0304-y

[S37] S. Zhao, H.-B. Zhang, J.-Q. Luo, Q.-W. Wang, B. Xu et al., Highly electrically conductive three-dimensional $Ti_3C_2T_x$ MXene/reduced graphene oxide hybrid aerogels with excellent electromagnetic interference shielding performances. ACS Nano **12**, 11193–11202 (2018). https://doi.org/10.1021/acsnano.8b05739

[S38] R. Liu, M. Miao, Y. Li, J. Zhang, S. Cao et al., Ultrathin biomimetic polymeric $Ti_3C_2T_x$ MXene composite films for electromagnetic interference shielding. ACS Appl. Mater. Interfaces **10**, 44787–44795 (2018). https://doi.org/10.1021/acsami.8b18347

[S39] R. Sun, H.-B. Zhang, J. Liu, X. Xie, R. Yang et al., Highly conductive transition metal carbide/carbonitride(MXene)@polystyrene nanocomposites fabricated by electrostatic assembly for highly efficient electromagnetic interference shielding. Adv. Funct. Mater. **27**, 1702807 (2017). https://doi.org/10.1002/adfm.201702807

[S40] M. Han, X. Yin, H. Wu, Z. Hou, C. Song et al., $Ti_3C_2T_x$ MXenes with modified surface for high-performance electromagnetic absorption and shielding in the x-band. ACS Appl. Mater. Interfaces **8**(32), 21011-21019 (2016). https://doi.org/10.1021/acsami.6b06455

[S41] H. Xu, X. Yin, X. Li, M. Li, S. Liang et al., Lightweight $Ti_3C_2T_x$ MXene/poly (vinyl alcohol) composite foams for electromagnetic wave shielding with absorption-dominated feature. ACS Appl. Mater. Interfaces **11**(10), 10198-10207 (2019). https://doi.org/10.1021/acsami.8b21671

[S42] B. Zhou, Z. Zhang, Y. Li, G. Han, Y. Feng et al., Flexible, robust, and multifunctional electromagnetic interference shielding film with alternating cellulose nanofiber and MXene layers. ACS Appl. Mater. Interfaces **12**(4), 4895-4905 (2020). https://doi.org/10.1021/acsami.9b19768

[S43] Z. Ma, S. Kang, J. Ma, L. Shao, Y. Zhang et al., Ultraflexible and mechanically strong double-layered aramid nanofiber– $Ti_3C_2T_x$ MXene/silver nanowire nanocomposite papers for high-performance electromagnetic interference shielding. ACS Nano **14**(7), 8368-8382 (2020). https://doi.org/10.1021/acsnano.0c02401

[S44] X. Li, X. Yin, S. Liang, M. Li, L. Cheng, L. Zhang, 2D carbide MXene $Ti_3C_2T_x$ as a novel high-performance electromagnetic interference shielding material. Carbon **146**, 210-217 (2019). https://doi.org/10.1016/j.carbon.2019.02.003

[S45] P. He, M.-S. Cao, Y.-Z. Cai, J.-C. Shu, W.-Q. Cao et al., Self-assembling flexible 2D carbide MXene film with tunable integrated electron migration and group relaxation toward energy storage and green EMI shielding. Carbon **157**, 80–89 (2020). https://doi.org/10.1016/j.carbon.2019.10.009

[S46] F. Xie, F. Jia, L. Zhuo, Z. Lu, L. Si et al., Ultrathin MXene/aramid nanofiber composite paper with excellent mechanical properties for efficient electromagnetic interference shielding. Nanoscale **11**, 23382–23391 (2019). https://doi.org/10.1039/c9nr07331k

[S47] C. Xiang, R. Guo, S. Lin, S. Jiang, J. Lan et al., Lightweight and ultrathin TiO_2-$Ti_3C_2T_x$/graphene film with electromagnetic interference shielding. Chem. Engin. J. **360**, 1158-1166 (2019). https://doi.org/10.1016/j.cej.2018.10.174

[S48] K. Raagulan, R. Braveenth, H.J. Jang, Y. Seon Lee, C.-M. Yang et al., Electromagnetic shielding by MXene-graphene-PVDF composite with hydrophobic, lightweight and flexible graphene coated fabric. Materials **11**(10), 1803 (2018). https://doi.org/10.3390/ma11101803

[S49] S.-J. Wang, D.-S. Li, L. Jiang, Synergistic effects between MXenes and Ni chains in flexible and ultrathin electromagnetic interference shielding films. Adv. Mater. Interfaces **6**, 1900961 (2019). https://doi.org/10.1002/admi.201900961

[S50] G.M. Weng, J. Li, M. Alhabeb, C. Karpovich, H. Wang et al., Layer-by-layer assembly of cross-functional semi-transparent MXene-carbon nanotubes composite films for next-generation electromagnetic interference shielding. Adv. Funct. Mater. **28**(44), 1803360 (2018). https://doi.org/10.1002/adfm.201803360

[S51] X. Jin, J. Wang, L. Dai, X. Liu, L. Li et al., Flame-retardant poly(vinyl alcohol)/MXene multilayered films with outstanding electromagnetic interference shielding and thermal conductive performances. Chem. Eng. J. **380**, 122475 (2020). https://doi.org/10.1016/j.cej.2019.122475

[S52] M. Han, X. Yin, K. Hantanasirisakul, X. Li, A. Iqbal et al., Anisotropic MXene aerogels with a mechanically tunable ratio of electromagnetic wave reflection to absorption. Adv. Opt. Mater. **7**(10), 1900267 (2019). https://doi.org/10.1002/adom.201900267

[S53] Z. Fan, D. Wang, Y. Yuan, Y. Wang, Z. Cheng et al., A lightweight and conductive MXene/graphene hybrid foam for superior electromagnetic interference shielding. Chem. Eng. J. **381**, 122696 (2020). https://doi.org/10.1016/j.cej.2019.122696

[S54] C. Weng, G. Wang, Z. Dai, Y. Pei, L. Liu et al., Buckled AgNW/MXene hybrid hierarchical sponges for high-performance electromagnetic interference shielding. Nanoscale **11**, 22804–22812 (2019). https://doi.org/10.1039/c9nr07988b

[S55] X. Wu, B. Han, H.-B. Zhang, X. Xie, T. Tu et al., Compressible, durable and conductive polydimethylsiloxane-coated MXene foams for high-performance electromagnetic interference shielding. Chem. Eng. J. **381**, 122622 (2020). https://doi.org/10.1016/j.cej.2019.122622

[S56] R. Bian, G. He, W. Zhi, S. Xiang, T. Wang et al., Ultralight MXene-based aerogels with high electromagnetic interference shielding performance. J. Mater. Chem. C **7**, 474–478 (2019). https://doi.org/10.1039/c8tc04795b

[S57] P. Sambyal, A. Iqbal, J. Hong, H. Kim, M.-K. Kim et al., Ultralight and mechanically robust $Ti_3C_2T_x$ hybrid aerogel reinforced by carbon nanotubes for electromagnetic interference shielding. ACS Appl. Mater. Interfaces **11**(41), 38046-38054 (2019). https://doi.org/10.1021/acsami.9b12550

[S58] Z. Zhou, J. Liu, X. Zhang, D. Tian, Z. Zhan et al., Ultrathin MXene/calcium alginate aerogel film for high-performance electromagnetic interference shielding. Adv. Mater. Interfaces **6**, 1802040 (2019). https://doi.org/10.1002/admi.201802040

[S59] M. Shen, J. Ni, Y. Cao, Y. Yang, W. Wang et al., Low infrared emitter from Ti_3C_2T MXene towards highly-efficient electric/solar and passive radiative heating. J. Mater. Sci. Technol. **133**, 32–40 (2023). https://doi.org/10.1016/j.jmst.2022.04.059

[S60] Salihoglu, H.B. Uzlu, O. Yakar, S. Aas, O. Balci et al., Graphene-based adaptive thermal camouflage. Nano Lett. **18**, 4541–4548 (2018). https://doi.org/10.1021/acs.nanolett.8b01746

[S61] M. Said Ergoktas, G. Bakan, P. Steiner, C. Bartlam, Y. Malevich et al., Graphene-enabled adaptive infrared textiles. Nano Lett. **20**(7), 5346-5352 (2020). https://doi.org/10.1021/acs.nanolett.0c01694

[S62] Y. Sun, Y. Wang, C. Zhang, S. Chen, H. Chang et al., Flexible mid-infrared radiation modulator with multilayer graphene thin film by ionic liquid gating. ACS Appl. Mater. Interfaces **11**, 13538–13544 (2019). https://doi.org/10.1021/acsami.8b21900

[S63] Z. Chen, K. Yang, T. Xian, C. Kocabas, S.V. Morozov et al., Electrically controlled thermal radiation from reduced graphene oxide membranes. ACS Appl. Mater. Interfaces **13**(23), 27278-27283 (2021). https://doi.org/10.1021/acsami.1c04352

[S64] S. Feng, Y. Yi, B. Chen, P. Deng, Z. Zhou et al., Rheology-guided assembly of a highly aligned MXene/cellulose nanofiber composite film for high-performance electromagnetic interference shielding and infrared stealth. ACS Appl. Mater. Interfaces **14**, 36060–36070 (2022). https://doi.org/10.1021/acsami.2c11292

[S65] L. Li, M. Shi, X. Liu, X. Jin, Y. Cao et al., Ultrathin titanium carbide (MXene) films for high-temperature thermal camouflage. Adv. Funct. Mater. **31**, 2101381 (2021). https://doi.org/10.1002/adfm.202101381

[S66] Y. Zhang, L. Li, Y. Cao, Y. Yang, W. Wang et al., High-strength, low infrared-emission nonmetallic films for highly efficient Joule/solar heating, electromagnetic interference shielding and thermal camouflage. Mater. Horiz. **10**, 235–247 (2023). https://doi.org/10.1039/d2mh01073a

[S67] Y. Li, C. Xiong, H. Huang, X. Peng, D. Mei et al., 2D $Ti_3C_2T_x$ MXenes: Visible black but infrared white materials. Adv. Mater. **33**(41), 2103054 (2021). https://doi.org/10.1002/adma.202103054

[S68] Li K., Li Z., Xiong Z., Wang Y., Yang H. et al., Thermal camouflaging MXene robotic skin with bio-inspired stimulus sensation and wireless communication. Adv. Funct. Mater. **32**, 2110534 (2022). https://doi.org/10.1002/adfm.202110534

[S69] M. Han, D. Zhang, A. Singh, T. Hryhorchuk, C.E. Shuck et al., Versatility of infrared properties of MXenes. Mater. Today **64**, 31-39 (2023). https://doi.org/10.1016/j.mattod.2023.02.024

[S70] Z.H. Liu, G.D. Ban, S.T. Ye, W.Y. Liu, N. Liu et al., Infrared emissivity properties of infrared stealth coatings prepared by water-based technologies. Opt. Mater. Express **6**, 3716 (2016). https://doi.org/10.1364/ome.6.003716

[S71] Y. Wang, H. Zhou, Y. Gao, Z. Zhang, S. Liu et al., Low-infrared-emissivity al@ SiO2/EPDM composite coating compatible with low dielectric loss and antistatic property. Infrared Phys. Techn. **121**, 104025 (2022). https://doi.org/10.1016/j.infrared.2022.104025

[S72] L. Chen, Z. Ren, X. Liu, K. Wang, Q. Wang, Infrared–visible compatible stealth based on Al-SiO_2 nanoparticle composite film. Opt. Commun. **482**, 126608 (2021). https://doi.org/10.1016/j.optcom.2020.126608

[S73] G. Wu, D. Yu, Preparation and characterization of a new low infrared-emissivity coating based on modified aluminum. Prog. Org. Coat. **76**, 107–112 (2013). https://doi.org/10.1016/j.porgcoat.2012.08.018

[S74] C. Shao, G. Xu, X. Shen, H. Yu, X. Yan, Infrared emissivity and corrosion-resistant property of maleic anhydride grafted ethylene-propylene-diene terpolymer (EPDM-g-MAH)/Cu coatings. Surf. Coat. Technol. **204**, 4075–4080 (2010). https://doi.org/10.1016/j.surfcoat.2010.05.036

[S75] H. Yu, G. Xu, X. Shen, X. Yan, C. Cheng, Low infrared emissivity of polyurethane/Cu composite coatings. Appl. Surf. Sci. **255**, 6077–6081 (2009). https://doi.org/10.1016/j.apsusc.2009.01.019

[S76] X. Yan, G. Xu, Effect of surface modification of Cu with ag by ball-milling on the corrosion resistance of low infrared emissivity coating. Mater. Sci. Engin.: B **166**(2), 152-157 (2010). https://doi.org/10.1016/j.mseb.2009.10.031

[S77] X. Ye, C. Zheng, X. Xiao, S. Cai. Synthesis, characterization and infrared emissivity study of SiO_2/Ag/TiO_2 “sandwich” core-shell composites. Mater. Lett. **141**, 191-193 (2015). https://doi.org/10.1016/j.matlet.2014.11.085

[S78] M. Li, D. Liu, H. Cheng, L. Peng, M. Zu Manipulating metals for adaptive thermal camouflage. Sci. Adv. **6**, eaba3494 (2020). https://doi.org/10.1126/sciadv.aba3494

[S79] L. Peng, D. Liu, H. Cheng, S. Zhou, M. Zu A multilayer film based selective thermal emitter for infrared stealth technology. Adv. Opt. Mater. **6**, 1801006 (2018). https://doi.org/10.1002/adom.201801006

[S80] B. V. Bergeron, K. C. White, J. L. Boehme, A. H. Gelb, P. B. Joshi. Variable absorptance and emittance devices for thermal control. J. Phys. Chem. C **112**(3), 832-838 (2008). https://doi.org/10.1021/jp076336d

[S81] Z. Huang, W. Zhou, X. Tang. Effects of annealing time on infrared emissivity of the pt film grown on ni alloy. Appl. Surface Sci. **256**(7), 2025-2030 (2010). https://doi.org/10.1016/j.apsusc.2009.09.042

[S82] M. Pan, Y. Huang, Q. Li, H. Luo, H. Zhu et al., Multi-band middle-infrared-compatible camouflage with thermal management via simple photonic structures. Nano Energy **69**, 104449 (2020). https://doi.org/10.1016/j.nanoen.2020.104449

[S83] Z. Huang, W. Zhou, X. Tang, D. Zhu, F. Luo. Effects of substrate roughness on infrared-emissivity characteristics of au films deposited on ni alloy. Thin Solid Films. **519**(10), 3100-3106 (2011). https://doi.org/10.1016/j.tsf.2010.12.157

[S84] J. Xu, M. Gao, L. Lu, Y. Wang, X. Liu, Study on the resistivity and infrared emissivity of TiNx films at different sputtering power. Infrared Phys. Technol. **119**, 103946 (2021). https://doi.org/10.1016/j.infrared.2021.103946

[S85] Y. Qin, M. Zhang, Y. Guan, X. Huang, Laser absorption and infrared stealth properties of Al/ATO composites. Ceramics Int. **45**(11), 14312-14315 (2019). https://doi.org/10.1016/j.ceramint.2019.04.144

[S86] Zhu H., Li Q., Zheng C., Hong Y., Xu Z. et al., High-temperature infrared camouflage with efficient thermal management. Light. Sci. Appl. **9**, 60 (2020). https://doi.org/10.1038/s41377-020-0300-5

[S87] X. Bu, Y. Zhou, M. He, Z. Chen, T. Zhang Optically active SiO_2/TiO_2/polyacetylene multilayered nanospheres: Preparation, characterization, and application for low infrared emissivity. Appl. Surf. Sci. **288**, 444–451 (2014). https://doi.org/10.1016/j.apsusc.2013.10.053

[S88] K. Tang, X. Wang, K. Dong, Y. Li, J. Li et al., A thermal radiation modulation platform by emissivity engineering with graded metal–insulator transition. Adv. Mater. **32**, 1907071 (2020). https://doi.org/10.1002/adma.201907071

[S89] H. Ji, D. Liu, H. Cheng, C. Zhang, L. Yang, Vanadium dioxide nanopowders with tunable emissivity for adaptive infrared camouflage in both thermal atmospheric windows. Solar Energy Mater. Solar Cells **175**, 96-101 (2018). https://doi.org/10.1016/j.solmat.2017.10.013

[S90] W. Zhang, D. Lv, Preparation and characterization of Ge/TiO_2 one-dimensional photonic crystal with low infrared-emissivity in the 8–14 μm band. Mater. Res. Bulletin **124**, 110747 (2020). https://doi.org/10.1016/j.materresbull.2019.110747

[S91] X. Wang, Y. Tang, Y. Wang, L. Ke, X. Ye et al., Leather enabled multifunctional thermal camouflage armor. Chem. Eng. Sci. **196**, 64–71 (2019). https://doi.org/10.1016/j.ces.2018.12.005

[S92] E. Sani, M. Meucci, L. Mercatelli, A. Balbo, C. Musa et al., Titanium diboride ceramics for solar thermal absorbers. Solar Energy Mater. Solar Cells. **169**, 313-319 (2017). https://doi.org/10.1016/j.solmat.2017.05.038

[S93] E. Sani, L. Mercatelli, D. Jafrancesco, J. Sans, D. Sciti, Ultra-high temperature ceramics for solar receivers: Spectral and high-temperature emittance characterization. J. European Opt. Soc. **7**, 12052 (2012). https://doi.org/10.2971/jeos.2012.12052

[S94] M. Benlattar, E.M. Oualim, M. Harmouchi, A. Mouhsen, A. Belafhal Radiative properties of cadmium telluride thin film as radiative cooling materials. Opt. Commun. **256**, 10–15 (2005). https://doi.org/10.1016/j.optcom.2005.06.033

[S95] T. Kim, J. Y. Bae, N. Lee, H.H. Cho, Hierarchical metamaterials for multispectral camouflage of infrared and microwaves. Adv. Funct. Mater. **29**(10), 1807319 (2019). https://doi.org/10.1002/adfm.201807319

[S96] W. Gu, J. Tan, J. Chen, Z. Zhang, Y. Zhao et al., Multifunctional bulk hybrid foam for infrared stealth, thermal insulation, and microwave absorption. ACS Appl. Mater. Interfaces **12**(25), 28727-28737 (2020). https://doi.org/10.1021/acsami.0c09202
